# Comparison of EEG Source Localization Using Simplified and Anatomically Accurate Head Models in Younger and Older Adults

**DOI:** 10.1109/TNSRE.2023.3281356

**Published:** 2023-06-13

**Authors:** Chang Liu, Ryan J. Downey, Yiru Mu, Natalie Richer, Jungyun Hwang, Valay A. Shah, Sumire D. Sato, David J. Clark, Chris J. Hass, Todd M. Manini, Rachael D. Seidler, Daniel P. Ferris

**Affiliations:** J. Crayton Pruitt Family Department of Biomedical Engineering, University of Florida, Gainesville, FL 32611 USA; J. Crayton Pruitt Family Department of Biomedical Engineering, University of Florida, Gainesville, FL 32611 USA; J. Crayton Pruitt Family Department of Biomedical Engineering, University of Florida, Gainesville, FL 32611 USA; J. Crayton Pruitt Family Department of Biomedical Engineering, University of Florida, Gainesville, FL 32611 USA.; Department of Kinesiology and Applied Health, University of Winnipeg, Winnipeg, MB R3B 2E9, Canada; Department of Physiology and Aging, University of Florida, Gainesville, FL 32611 USA; Department of Applied Physiology and Kinesiology, University of Florida, Gainesville, FL 32611 USA; Department of Applied Physiology and Kinesiology, University of Florida, Gainesville, FL 32611 USA; Department of Physiology and Aging, University of Florida, Gainesville, FL 32611 USA,; Brain Rehabilitation Research Center at the Malcom Randall VA Medical Center, Gainesville, FL 32608 USA; Department of Applied Physiology and Kinesiology, University of Florida, Gainesville, FL 32611 USA; Department Health Outcomes & Biomedical Informatics, University of Florida, Gainesville, FL 32611 USA; Department of Applied Physiology and Kinesiology, University of Florida, Gainesville, FL 32611 USA; J. Crayton Pruitt Family Department of Biomedical Engineering, University of Florida, Gainesville, FL 32611 USA

**Keywords:** EEG, forward modeling, head model, source localization

## Abstract

Accuracy of electroencephalography (EEG) source localization relies on the volume conduction head model. A previous analysis of young adults has shown that simplified head models have larger source localization errors when compared with head models based on magnetic resonance images (MRIs). As obtaining individual MRIs may not always be feasible, researchers often use generic head models based on template MRIs. It is unclear how much error would be introduced using template MRI head models in older adults that likely have differences in brain structure compared to young adults. The primary goal of this study was to determine the error caused by using simplified head models without individual-specific MRIs in both younger and older adults. We collected high-density EEG during uneven terrain walking and motor imagery for 15 younger (22±3 years) and 21 older adults (74±5 years) and obtained T_1_-weighted MRI for each individual. We performed equivalent dipole fitting after independent component analysis to obtain brain source locations using four forward modeling pipelines with increasing complexity. These pipelines included: 1) a generic head model with template electrode positions or 2) digitized electrode positions, 3) individual-specific head models with digitized electrode positions using simplified tissue segmentation, or 4) anatomically accurate segmentation. We found that when compared to the anatomically accurate individual-specific head models, performing dipole fitting with generic head models led to similar source localization discrepancies (up to 2 cm) for younger and older adults. Co-registering digitized electrode locations to the generic head models reduced source localization discrepancies by ~6 mm. Additionally, we found that source depths generally increased with skull conductivity for the representative young adult but not as much for the older adult. Our results can help inform a more accurate interpretation of brain areas in EEG studies when individual MRIs are unavailable.

## I. Introduction

Electroencephalography (eeg) source localization relies on accurate forward head models to parse electrode channel level signals into brain source signals. Blind source separation approaches like Independent Component Analysis (ICA) can identify brain signals contributing to recorded scalp electrode potentials [1]. Source localization identifies the brain locations that generate the separated electrical brain signals [2], [3]. One important factor contributing to the accuracy of EEG source localization is the volume conduction of the head when using forward modeling [4]. Volume conduction head models are described by head anatomy and tissue electrical properties. Head models with realistic modeling of anatomical structures can substantially improve the accuracy of EEG source localization based on many simulation studies [5], [6], [7], [8], [9], [10]. Additionally, a previous analysis of young adults has shown that simplified head models (e.g., spherical head models) have larger source localization errors when compared with more realistically shaped head models based on magnetic resonance images (MRIs) [4]. However, it has yet to be investigated how source localization results differ between using simplified head models and anatomically accurate head models in older adults. It is plausible to expect that errors might be greater in older adult participants than in younger adults, given the brain atrophy that occurs with aging and the increased distance between the skull and cortex.

One common approach to performing EEG source localization is equivalent dipole fitting [1], [11]. This approach first performs independent component analysis of the EEG signal to identify: 1) the independent components whose time series are maximally independent of each other, and 2) a weight matrix describing the projection from each of the independent components to the scalp electrodes [12], [13]. The independent components from brain sources have scalp projections that closely resemble the projection of a single equivalent brain dipole [1]. The locations of each brain source can then be estimated using dipole fitting to identify the dipole positions and orientations that can best describe the scalp projection of the independent components.

There is a need to compare source localization results using generic head models based on template MRIs versus using individual-specific head models from individual MRIs. Despite the benefits of using anatomically accurate head models for EEG source localization, the acquisition of individual MRIs can be costly and may not be feasible for all EEG studies due to many reasons, such as a lack of access to an MR scanner. Creating individual head models is also computationally expensive. As a result, it is common to use generic head models available in open-source software packages such as EEGLAB [14] to perform source localization [15], [16]. These generic head models are usually created based on the template MRIs (e.g., ICBM 152 [17]) from young adults, which may not provide the best fit for data from older adult participants.

Because prior head model comparisons have relied exclusively on young adults, it is necessary to evaluate the effect of head models on source localization in older adults. Morphological changes in the aging brain can influence the electrical properties of the head model during forward modeling [18], [19], [20]. Age-related changes include a decrease in total brain mass [21], cortical thinning [22], cerebrospinal fluid expansion [18], ventricles enlargement [23], and gyral atrophy [24], [25]. These age differences in brain structure can influence the electrical properties of the head model during forward modeling. For example, cerebrospinal fluid volume increases in ventricles and between the cortex and the skull due to overall atrophy with aging. Since the cerebrospinal fluid is more conductive than the skull and the brain, increased cerebrospinal fluid shunts more current and attenuates scalp potentials for older adults compared to younger adults [19], [26], [27]. While these prior studies investigated the effects of age differences in brain structure on EEG forward modeling, further work is needed to examine how these changes may affect EEG source localization.

Another important factor affecting the EEG source localization estimations is the electrode locations on the scalp [4]. Electrode location shifting by 5° could result in about 10 mm localization error [4], [28]. Using a structural scanner to digitize electrode locations and fiducial locations can improve the co-registering of the electrodes to the head model with high reliability and low variability [29]. These digitized electrode locations can then be aligned to the individual-specific head model or the generic head model to perform EEG source localization.

Skull conductivity also has marked effects on head model modeling and EEG source localization [30], [31], [32]. A large range of skull conductivity has been reported [33], [34], [35], [36], [37], [38]. For example, a recent review paper by McCann et al. reports the estimates of skull conductivity to range from 0.0008 S/m to 0.289 S/m [26]. The uncertainty of skull conductivity values could lead to large variations in source locations by centimeters [32]. Since aging is associated with lower skull conductivity [39], [40] and the ground truth of skull conductivity is unknown, understanding how skull conductivity affects the estimated source location is important for interpreting brain activities in EEG studies of older adults.

In this study, we aimed to quantify the effect of using simplified head models on source localization using dipole fitting in both younger and older participants. Previous literature suggests that individual-specific MRIs and head models produce the most accurate localization of source locations [4]. However, obtaining individual-specific MRIs for source localization may not be possible in many instances. Thus, the primary goal of this study was to determine the error caused by using simplified head models without individual-specific MRIs in both younger and older adults. We hypothesized that simpler head models would have larger source localization discrepancies compared to the most anatomically accurate head model. We also hypothesized that source localization differences between simplified generic head models and the most anatomically accurate head model would be higher in older adults versus younger adults because of larger variations in older adult anatomy compared to commonly used MRI templates. To perform a thorough analysis of forward modeling error on source location estimations, we further quantified source localization discrepancies introduced by inexact electrode locations, inaccurate brain region segmentation, and variations in skull conductivity. We collected high-density EEG data on both younger and older adults performing uneven terrain walking and motor imagery tasks and obtained a T1-weighted MRI for each participant. We performed equivalent dipole fitting after independent component analysis to obtain brain source locations using four forward modeling pipelines with increasing complexity. These pipelines included: 1) a generic head model with template electrode positions or 2) digitized electrode positions, 3) individual-specific head models with digitized electrode positions using simplified tissue segmentation, or 4) anatomically accurate segmentation. Additionally, we conducted a sensitivity analysis to assess how skull conductivity affects source locations for three younger and three older participants. These results will provide a better understanding of the factors that affect EEG source localization and enable a more accurate interpretation of brain activation in the older adult population for EEG studies.

## II. Methods

### A. Participants

We analyzed a dataset with 15 younger adult participants and 21 older participants from the ongoing Mind in Motion study (Table I) [41]. Full inclusion and exclusion criteria were reported by Clark et al. [41]. Participants were excluded for self-reported neurological or severe cardiovascular, orthopedic, or psychiatric diagnoses. Participants were also excluded if they scored <23 on the Montreal Cognitive Assessment (MoCA). All participants provided informed consent before participating. The study was conducted in accordance with the Declaration of Helsinki and approved by the Institutional Review Board of the University of Florida (IRB 201802227).

### B. Experimental Protocol

The experimental protocol is a subset of the larger study (Mind in Motion, NCT03737760). Details of the full protocol were provided in [41]. The experiment protocol included one session of EEG assessments and one session of MRI scans. The two sessions were conducted on separate days within approximately 30 days or less of each other. Two of the older adults were excluded from further analysis because they had an MRI scan more than one year after their first EEG visit due to the COVID-19 pandemic in 2020. Three of our MRI scans for younger adults were collected about six months to a year later than the EEG visit due to the same reason. Since there is usually no substantial brain structural change occurring within six months in younger adults [42], this minor brain structural change would very unlikely impact our analysis with younger adults. Thus, we kept the three younger adults in our analysis. During the EEG session, participants completed trials including treadmill walking on four different levels of uneven terrain (flat, low, medium, high) and at different walking speeds (0.25m/s, 0.5m/s, 0.75m/s, and 1.00 m/s). The order of the trials was randomized. Details of the study design were reported in the previous papers [41] and [43]. Participants completed two trials per condition, and each trial lasted for 3 minutes. They also completed a 3-min resting trial and a 10-min seated motor imagery trial. During the motor imagery trial, participants imagined completing different levels of uneven terrain per instructions on the computer monitor. In total, there were approximately 60 minutes of EEG data for each participant.

During this visit, participants wore a custom-made dual-layer EEG cap (ActiCAP snap sensors; Brain Products GmbH, Germany) [44], [45], including 120 scalp electrodes and 120 noise electrodes. The scalp electrodes followed a 10-05 electrode system. We inverted and mechanically coupled noise electrodes to the scalp electrodes [44], [45]. We used the data from noise electrodes to help remove artifacts during EEG processing. We used a conductive fabric to bridge the noise electrodes as an artificial skin circuit. We re-purposed eight of the original 128 scalp electrodes (TP9, P9, PO9, O9, O10, PO10, P10, and TP10) to measure the muscle activity at sternocleidomastoid and trapezius on the left and right sides [45]. We aimed to keep scalp electrode impedance values below 15kΩ during the setup. We digitized the electrode locations using a structural scanner (ST01, Occipital Inc., San Francisco, CA). We used four LiveAmp 64 amplifiers and logged EEG data at 500Hz. The online reference and ground electrodes were at CPz and Fpz, respectively.

### C. MRI Acquisition

On a separate day, we collected structural MRIs for participants. We obtained the anatomical brain structure from a T_1_-weighted sequence. The parameters for this anatomical image were: repetition time (TR) = 2000 ms, echo time (TE) = 2.99 ms, flip angle = 8°, voxel resolution = 0.8 mm^3^, field of view = 256 × 256 × 167 mm^2^ (4:22 minutes of scan time), using a 64-channel coil array on a 3T Siemens MAGNETOM Prisma Magnetic Resonance scanner.

### D. Data Processing

#### 1) Magnetic Resonance Imaging Processing:

We processed the T_1_-weighted MRIs using Fieldtrip (v.20210910) for each participant. The images were resliced to be isotropic (1 mm^3^). We digitized the fiducial locations (left/right preauricular, nasion) on the MRIs. To compute the gray matter volume from the T_1_-weighted image, we used the Computational Anatomy Toolbox (CAT12, version r1278). We used the default CAT12 preprocessing steps to obtain the whole-brain gray matter mask and ventricle mask for each participant. We also obtained the total intracranial volume for each participant to normalize the gray matter and ventricular volumes (Table I).

#### 2) EEG Processing:

**Fig. 1.**
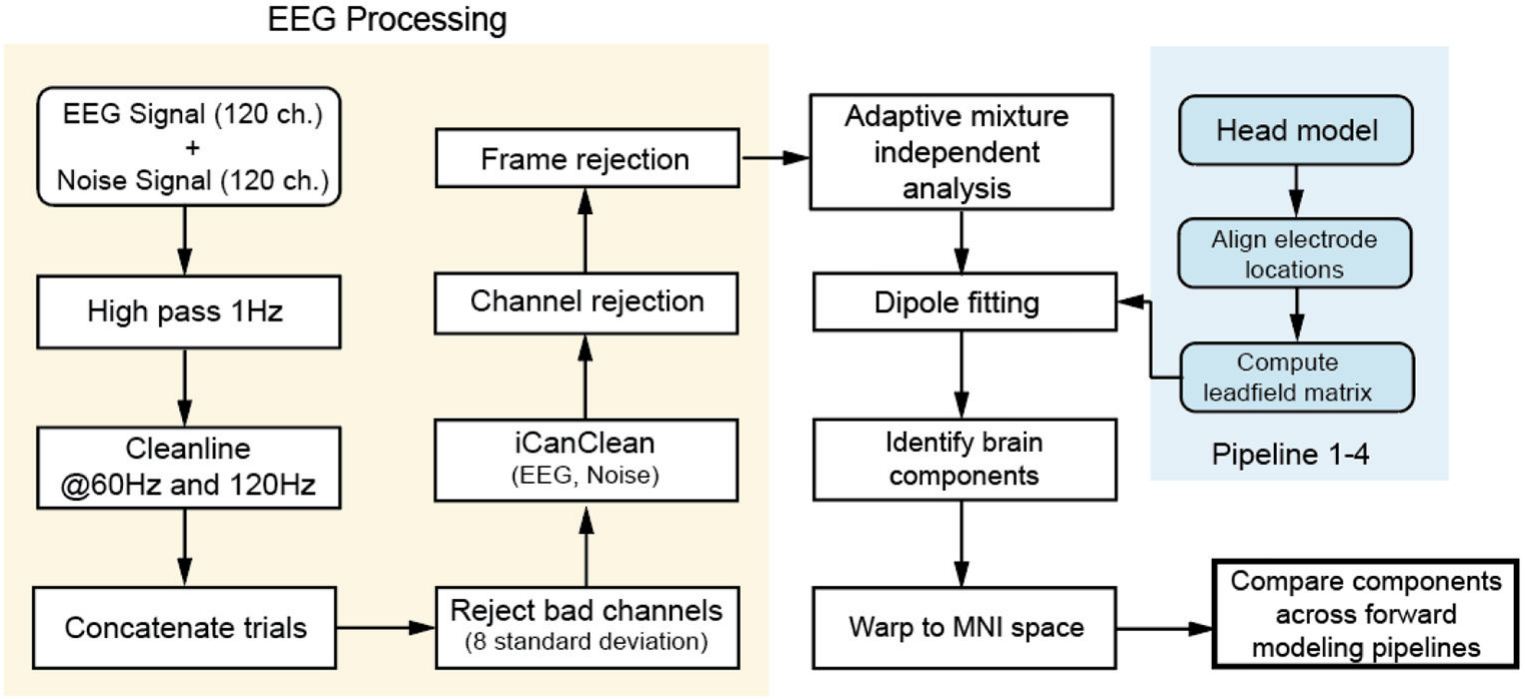
Data processing flowchart with steps for EEG pre-processing, forward modeling, and source localization. Blue blocks indicate steps for forward modeling. We constructed four forward modeling pipelines 1-4 with increasing levels of complexity. Lastly, we warped the brain source locations to Montreal Neurological Institute (MNI) space to allow for inter-subject comparisons across forward modeling pipelines.

We processed all EEG data using custom Matlab scripts (R2021b) and EEGLAB (v 2021.0) [14](Fig. 1). We first applied a 1Hz (−6dB at 0.5Hz) high-pass filter with a zero-phase, finite impulse response (*eegfiltnew*) on all scalp, noise, and muscle channels to remove drift for each trial. We used the CleanLine plugin in EEGLAB to remove line noise at 60 Hz and 120 Hz. We then merged all trials. We rejected bad channels that were eight standard deviations away from the mean of EEG and noise channels, respectively. We then used iCanClean [46] to remove the EEG artifacts that highly correlated with noise electrodes (rho^2^ = 0.9 with a two-second moving window). We used *clean_artifacts* with default parameters in EEGLAB to identify bad channels and noisy time frames except for the following parameters (chan_crit1 = 0.5, win_crit1 = 0.4, winTol = [10, -Inf]). These parameters were selected in a preliminary analysis of a subset of the data, which aimed to minimize the number of channels and time frames rejected while maximizing a good number of brain components by ICLabel (Supplementary Fig. 1) [47]. We retained 116 [min = 102, max = 120] channels and rejected a maximum of 10% of time frames. We used adaptive mixture independent analysis (AMICA) [48] to decompose the preprocessed EEG data into statistically independent components. We later used the independent components to perform source localization.

### E. EEG Forward Modeling

**Fig. 2.**
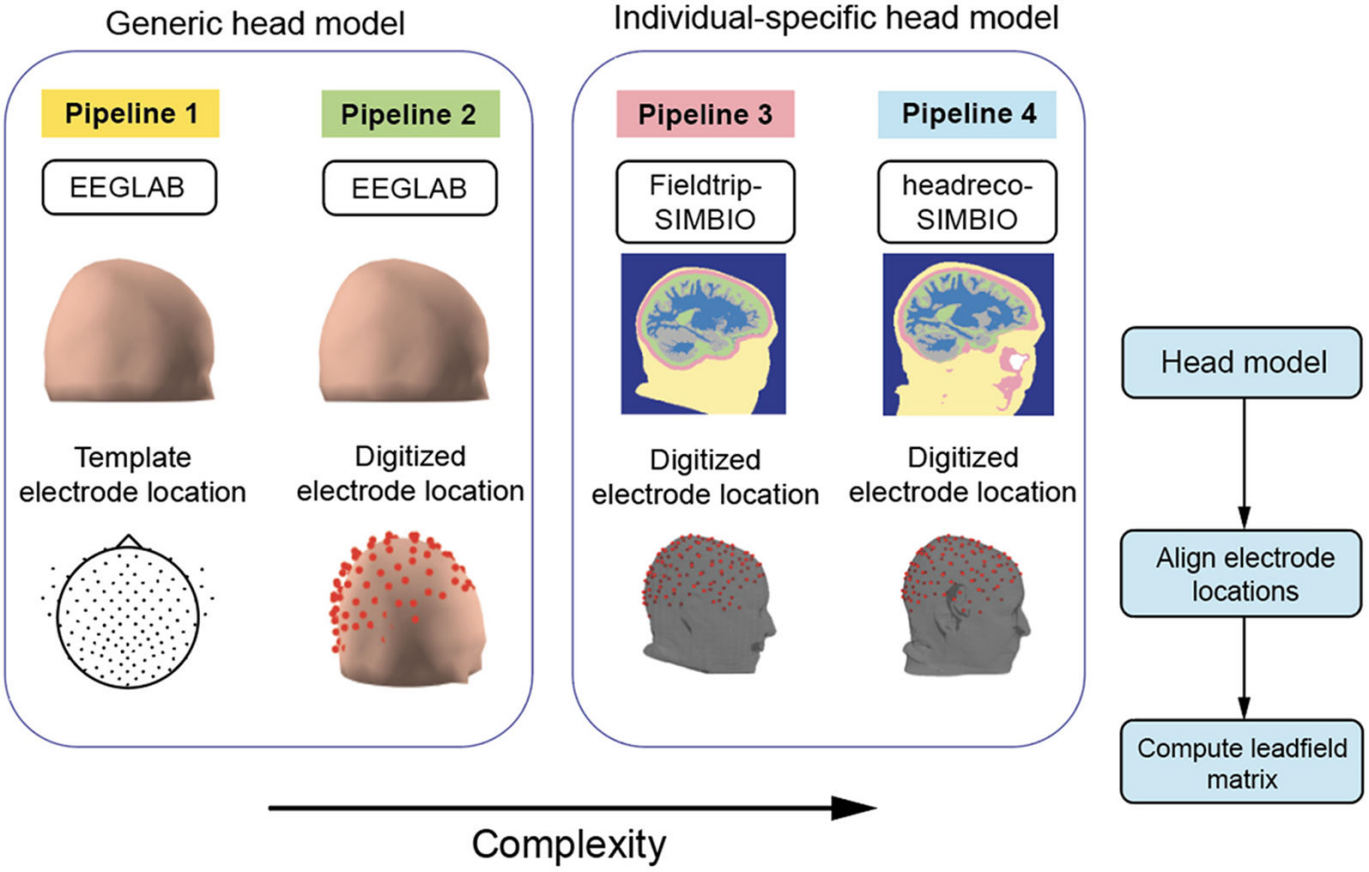
Four forward modeling pipelines with increasing levels of complexity. Pipelines included steps that created the head model mesh, aligned electrode locations to the generic head model or individual-specific head model, and computed leadfield matrix. Pipeline 1-2 used an available three-layer generic head model in EEGLAB. Pipeline 1 aligned template electrode positions to the generic head model. Pipeline 2 aligned digitized electrode positions to the generic head model. Pipeline 3-4 created individual-specific head models based on individual MRIs. Pipeline 3 used Fieldtrip-SIMBIO to perform simplified tissue segmentation when creating the head models. Pipeline 4 used headreco toolbox to perform anatomically accurate tissue segmentation when creating the head models.

We constructed four forward modeling pipelines, numbered 1-4, with increasing levels of complexity (Fig. 2). Each pipeline included steps to create the head model mesh, align electrode locations to the head model, and compute the leadfield matrix. Pipelines 1 and 2 used the three-layer boundary element generic head model in EEGLAB DIPFIT toolbox (v 4.3). This three-layer head model was based on the ICBM152 brain template in the Montreal Neurological Institute (MNI) coordinate from a young adult population (25 ± 5yrs) [17]. The conductivity values for each tissue type were as follows: gray matter: 0.33 S/m, scalp: 0.33 S/m, and skull: 0.0042 S/m [1] (Table II).

Pipelines 3 and 4 created individual-specific finite element head models based on individual MRIs. The conductivity values used for each tissue type for the individual-specific head models were as follows: gray matter: 0.33 S/m; white matter: 0.126 S/m; cerebrospinal fluid: 1.65 S/m; scalp: 0.33 S/m; air: 2.5 × 10^−14^S/m [49], [50] (Table II). We examined three different skull conductivity values for the individual-specific head models: 0.0042 S/m, 0.01 S/m, and 0.02 S/m. We used these skull conductivity values because 0.0042 S/m was consistent with the generic head model in EEGLAB [51], [52], 0.01 S/m was a commonly used conductivity value for the skull [30], [53], and 0.02 S/m was a suggested skull conductivity value in a recently published meta-analysis [26] (Table II). The conductivity values for gray matter and scalp are chosen to be consistent with the generic head model in the DIPFIT toolbox. We did not include anisotropic conductivity. Below are details about each pipeline.

#### 1) Pipeline 1:

We used the three-layer generic head model in EEGLAB DIPFIT toolbox. We aligned the generic electrode locations (10-05 Template) to the head model.

#### 2) Pipeline 2:

We used the three-layer generic head model in EEGLAB DIPFIT toolbox, similar to Pipeline 1. However, for Pipeline 2, we aligned the digitized electrode locations to the generic head model for each participant, rather than assume generic electrode locations. We obtained the participant-specific electrode locations using a 3D structural scan of the participant’s head using *getchanloc* (v 3.01) toolbox in EEGLAB. We co-registered the digitized electrode locations to the generic head model by first aligning to fiducial locations (left/right preauricular, nasion), and then we resized the electrode locations to best align on the generic head model.

#### 3) Pipeline 3:

**Fig. 3.**
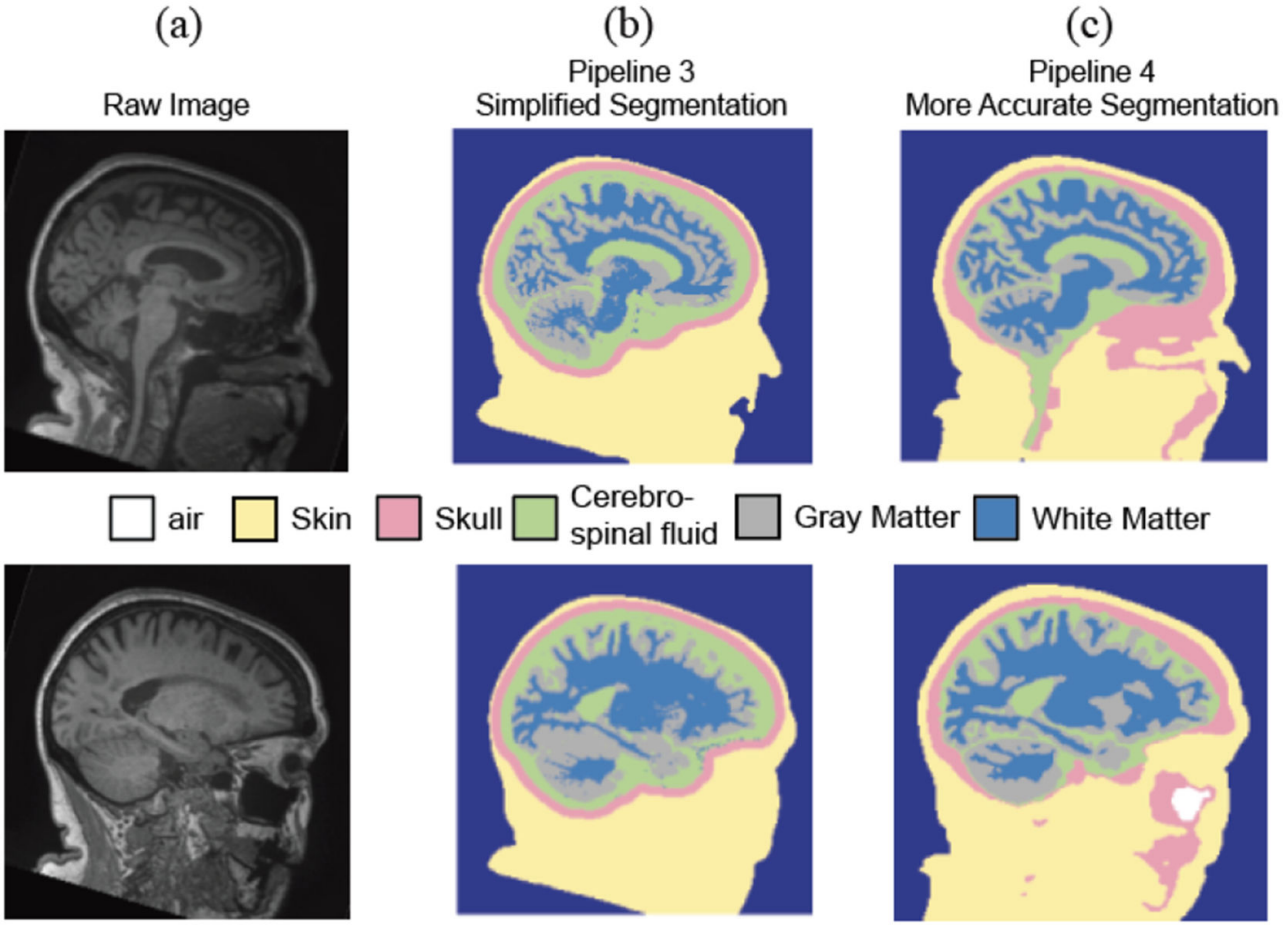
Example of tissue segmentation using Pipeline 3 and Pipeline 4. (a - left column) Raw MRI of one representative participant. Top row and bottom row represent two different slices of the MRI. (b - middle column) Pipeline 3 segmented the image into five tissue layers, including scalp, skull, cerebrospinal fluid, gray matter, and white matter. (c – right column) Pipeline 4 segmented the image into six tissue layers, including air, scalp, skull, cerebrospinal fluid, gray matter, and white matter. Note the marked difference in skull segmentation between the two pipelines as Pipeline 3 assumed a constant skull thickness.

We followed the Fieldtrip-SIMBIO pipeline to create the individual-specific head model [53]. We used the default settings in *ft_volumesegment* to segment individual MRIs into five tissue layers (scalp, skull, cerebrospinal fluid, gray matter, and white matter; Fig. 3a,b). Hexahedral meshes were generated with recommended node-shift parameters using *prepare_mesh_hexahedral*. We co-registered digitized electrode locations to the individual-specific head model by aligning the fiducial locations digitized in the MRIs to those in the structural scan. We calculated the leadfield matrix for each individual-specific head model using the SIMBIO toolbox in Fieldtrip. We distributed source positions in the gray matter 5 mm apart.

#### 4) Pipeline 4 (Most Anatomically Accurate):

The difference between Pipeline 4 and Pipeline 3 was that we used the headreco toolbox (v 3.2) to perform more accurate tissue segmentation in Pipeline 4 [54]. Here, individual MRIs were segmented into six tissue layers (scalp, skull, air, cerebrospinal fluid, gray matter, and white matter) (Fig. 3a,c). The rest of the steps were the same as in Pipeline 3. Similarly, we co-registered the electrode locations to the individual-specific head model by aligning the fiducial locations and computed the leadfield matrix using SIMBIO. We distributed source positions in the gray matter 5 mm apart.

### F. Source Localization

We fit each independent component with an equivalent dipole using *ft_dipolefitting* function in the Fieldtrip toolbox. The function worked by placing dipolar sources at different positions and orientations within the gray matter until it found the best dipolar source to optimally explain the measured data (i.e. to minimize the difference between forward modeled scalp topography and the measured data). We normalized individual MRIs to the MNI template using *ft_volumenormalise* which used SPM 12 with default settings to compare the dipole locations fitted across the forward modeling pipelines. We visually inspected the normalization results. We obtained the transformation matrix from the normalization and warped the dipole locations to the MNI template.

### G. Identify Brain Components

We retained brain components using the following criteria: 1) ICLabel [47](version: lite) classified the brain probability of greater or equal to 50%, 2) negative slope of the power density spectrum for 2-40 Hz to remove muscle components, 3) residual variance of dipole fitting <15%, 4) minimal high-frequency power coupling using PowPowCAT toolbox to further remove muscle components [55], and lastly, we visually inspected all the components and removed non-brain components.

### H. Comparison of Source Localization Across Pipelines

We used two metrics to compare the source localization results across pipelines. First, we computed the residual variance of the dipole fitting by calculating the difference between the modeled scalp topography and the measured data, normalized to the measured data. Residual variance measured the goodness of fit of the dipole fitting. Residual variance (%) was between 0% (perfectly matched) and 100% (the most dissimilar).

Second, we calculated the Euclidean distance between the dipole locations fitted by Pipelines 1-3 and that by fitted by Pipeline 4 (i.e., the most anatomically accurate) to understand how dipole locations varied across forward modeling pipelines.

### I. Sensitivity Analysis of Skull Conductivity Values

We performed a sensitivity analysis on how the estimated source locations changed with skull conductivity using Pipeline 4 for three young adults and three older adults [32], [56]. These participants were randomly selected from participants who had more than ten brain components. Due to the long computation time (~2 weeks/person), we could not perform the sensitivity analysis on the whole dataset. We focused our analysis on skull conductivity values because multiple studies have highlighted the effect of skull conductivities on the forward head models and source localization [30], [32]. We varied the skull conductivity values in the range of 0.0016 – 0.033 S/m in 100 steps [32], [56]. We computed the source depth as the minimum distance of the source location to the skull surface for each dipole as described in previous literature [32], [57].

### J. Statistical Analysis

All statistical analyses were performed in Matlab 2021b (Mathworks). We first compared the demographics and brain structure (gray matter volume and ventricle volume) metrics between the two age groups using two-sample t-tests. In cases where the normality assumption for the t-test was violated as determined by Shapiro-Wilk’s test, we conducted nonparametric Wilcoxon rank-sum tests to examine age group differences. We conducted a Pearson chi-square test to test for differences in the sex distribution within each age group.

We used linear mixed-effect models to examine the relationship between the outcome measures (residual variance and Euclidean distance) and independent variables pipelines and age groups. For residual variance, we included the main effects for Pipeline (1, 2, 3, 4) and Age group (younger and older) and the interaction between Pipeline and Age in the model. The reference level was Pipeline 4 and younger adults. For Euclidean distance, since we computed Euclidean distances relative to dipole locations estimated by Pipeline 4, we included the main effects for Pipeline (1, 2, 3) and Age group (younger and older) and the interaction between Pipeline and Age. The linear mixed-effect models were fit for each outcome measure at each skull conductivity value used for individual-specific head models (0.0042S/m, 0.01S/m, 0.02S/m). For all models, we included a random intercept to account for unmodeled sources of between-subject variability. Post-hoc comparisons were adjusted using the Bonferroni test. All significance levels were set to alpha <0.05.

## III. Results

### A. Effects of Head Model Simplicity on Source Localization

The number of brain components was similar for younger adults and older adults (13 ± 5 versus 11 ± 5, t(25) = 1.0, p = 0.30) using Pipeline 4. We only retained brain components for further analysis.

**Fig. 4.**
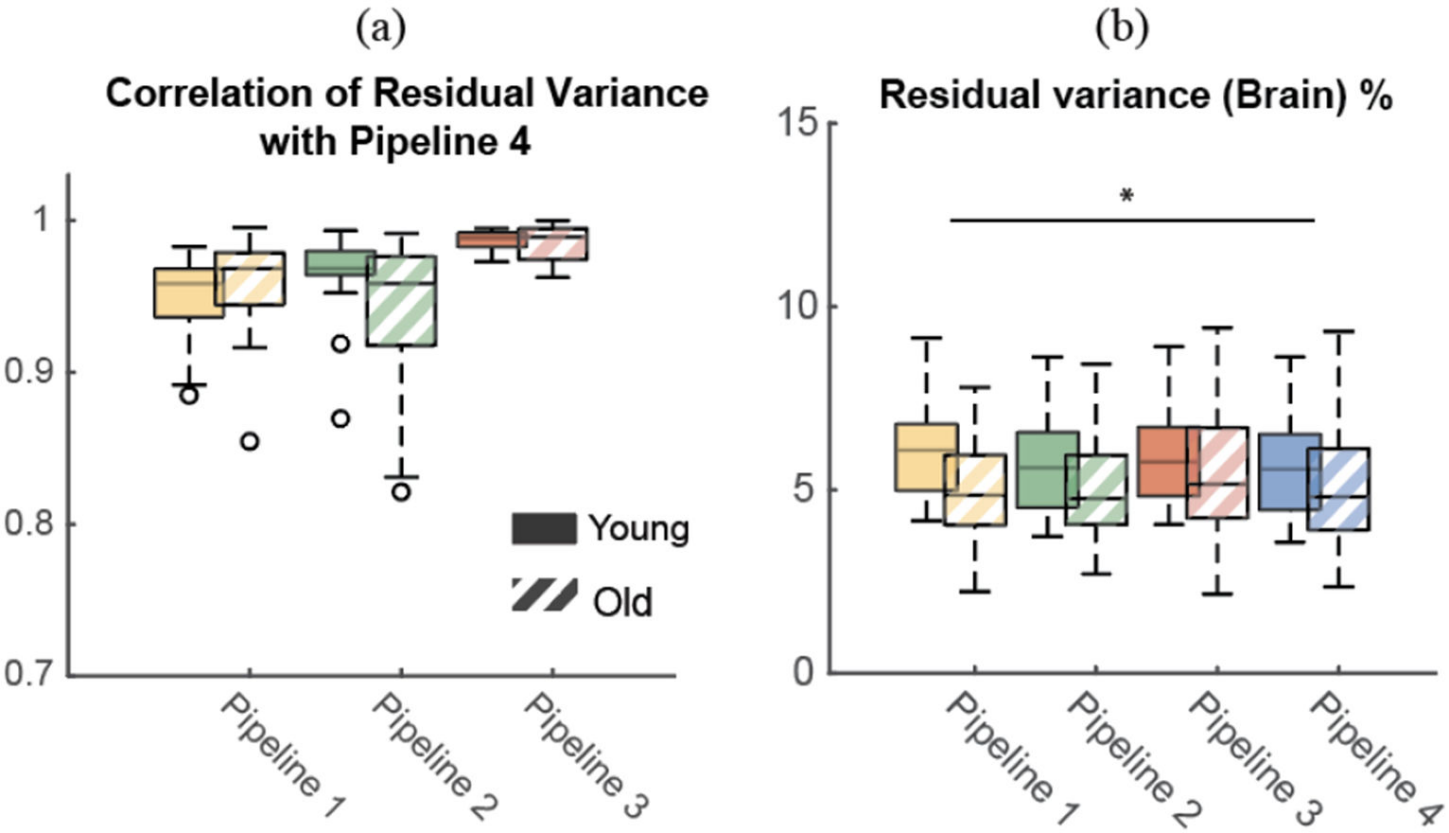
Boxplot of (a) correlation of residual variance between pipelines across participants and (b) residual variance of brain components (n = 15 younger adults; n = 19 older adults). Skull conductivity value was 0.01 S/m for the individual-specific head models. (a) Correlation of residual variances of brain components from Pipeline 1-3 with the most anatomically accurate Pipeline 4 across participants. (b) Median residual variance of brain components for each pipeline across participants. Solid: younger adults; Stripes: older adults. (* p < 0.05).

Residual variances for brain components from Pipelines 1-3 were highly correlated with the residual variances from Pipeline 4 across all participants (median [interquartile] r = 0.96 [0.03], r = 0.97 [0.05], r = 0.99 [0.01]; Fig. 4a). There was a significant effect of Pipeline on residual variance (F(1,128) = 3.74, p = 0.013), but there was no effect of Age (F(1,128) = 0.92, p = 0.34; Fig. 4b). At a group level, residual variances for Pipeline 1 were statistically higher than Pipeline 4 (5.23% [2.0%] vs. 5.17% [2.2%], p = 0.023), but the difference between the two pipelines was small.

**Fig. 5.**
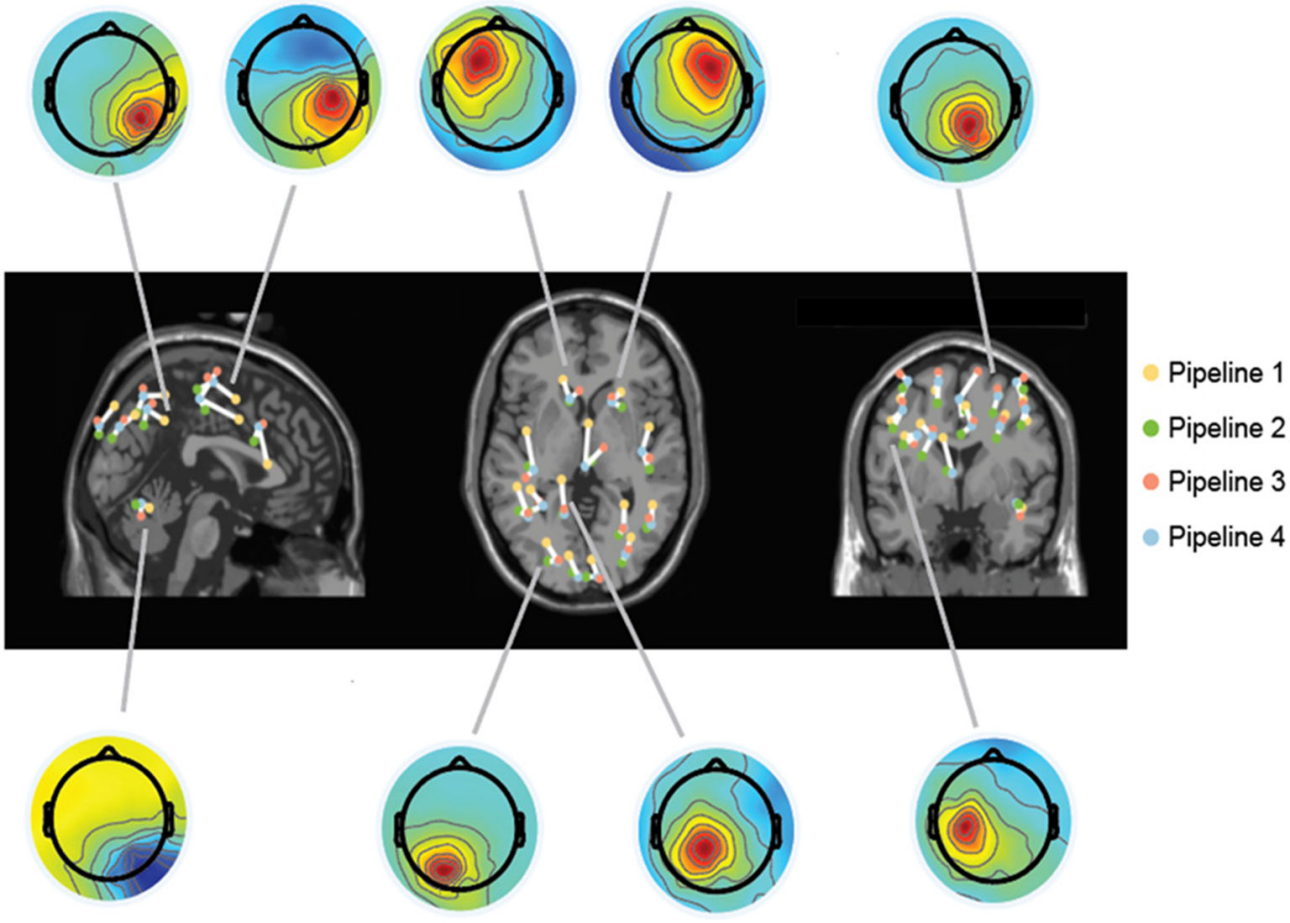
Dipole locations estimated by four forward modeling pipelines with increasing complexity warped to a template MRI in MNI space and representative brain components for one young participant. Yellow dots: dipoles estimated using Pipeline 1; Red: Pipeline 2; Green: Pipeline 3; and Blue: Pipeline 4. Dipoles that were grouped together were estimated using the same brain component.

**Fig. 6.**
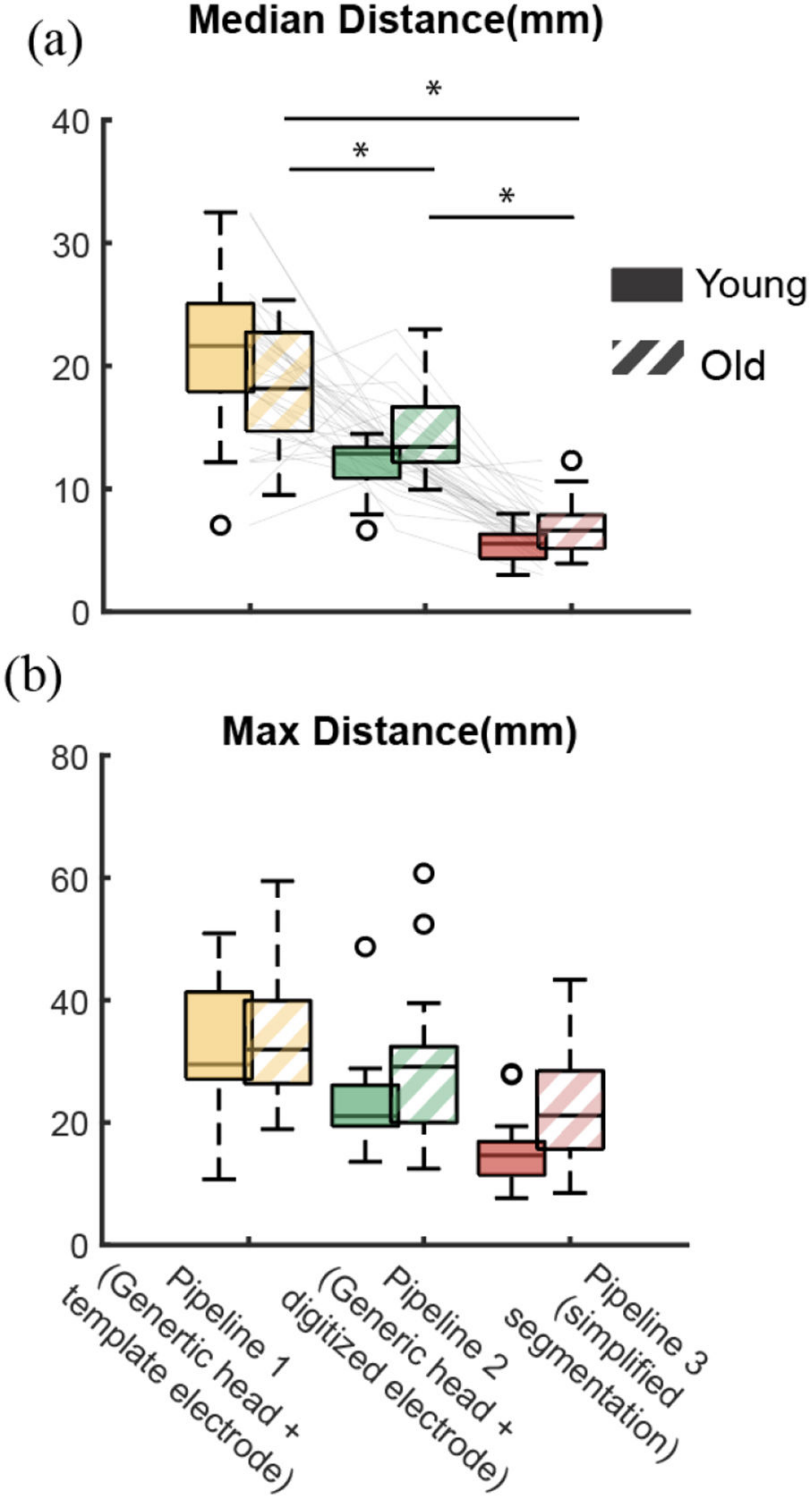
Median (a) and maximum (b) Euclidean distances between dipole fitted following Pipeline 1-3 and the reference Pipeline 4 across participants (n = 15 younger adults; n = 19 older adults). Skull conductivity value was 0.01 S/m for the individual-specific head models. Solid: younger adults; Stripes: older adults. Statistical comparisons were performed for median distances (a) only. (*p < 0.001).

Using simplified head models and template electrode locations during forward modeling demonstrated large localization discrepancies compared to the anatomically accurate reference Pipeline 4 (Fig. 5, Supplementary Fig. 2 for all participants). We only reported statistical results here for 0.01 S/m skull conductivity to improve readability as the statistical results when using other skull conductivities were similar (Supplementary Table 1). In general, there were large discrepancies between the dipole location estimated using Pipeline 1 and Pipeline 4 in the anteroposterior direction. We found a significant effect of Pipeline on Euclidean distances between dipoles (F(1,96) = 67.0, p < 0.001). The Euclidean distances between Pipelines 1-3 and the most anatomically accurate Pipeline 4 (median [interquartile]) were 19.1 [8.1] mm, 13.1 [2.8] mm, and 5.7 [2.3] mm when skull conductivity was 0.01 S/m for the individual-specific head models (Fig. 6a). Using template electrode locations with the generic head model increased the localization discrepancies by 6 mm compared to using the digitized electrode locations (Pipeline 1 vs. Pipeline 2; t(96) = −6.8, p < 0.001).

The maximum localization discrepancies referenced to Pipeline 4 were 31.5 [13.3] mm, 24 [10.5] mm, and 17 [10.5] mm for Pipelines 1-3, respectively (Fig. 6b). We did not perform statistical analysis for maximum discrepancies as the analysis was not included in our hypothesis.

### B. Age-Related Effects on Localization Difference

**Fig. 7.**
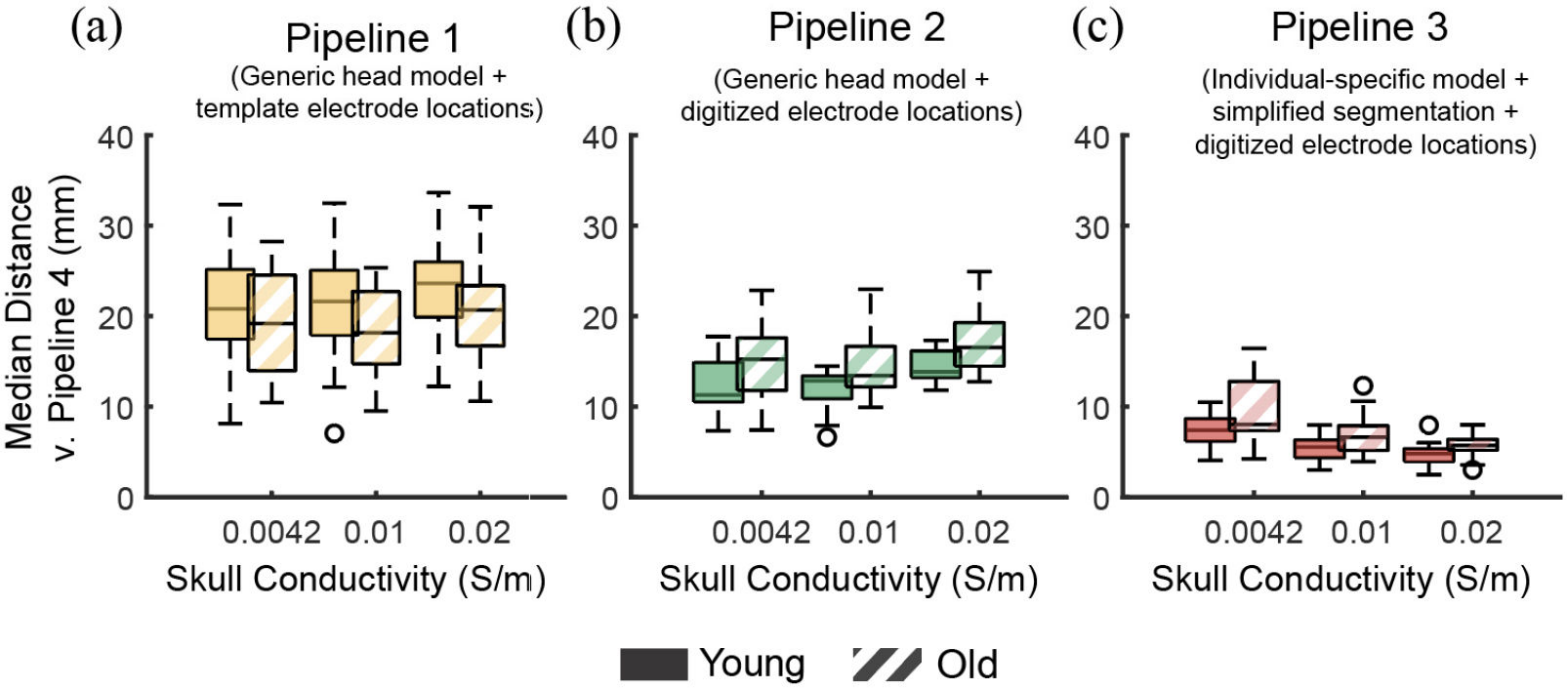
Median Euclidean distances between dipoles fitted following Pipeline 1 (a), Pipeline 2 (b), and Pipeline 3 (c) and the reference Pipeline 4 across participants (n = 15 younger; n = 19 older adults) when the skull conductivity was 0.0042S/m, 0.01S/m, and 0.02S/m for the individual-specific head models. Solid: younger adults; Stripes: older adults.

There was no difference in source localization discrepancies between the simplified head model and the most anatomically accurate model in older adults versus younger adults regardless of the skull conductivity (Fig. 7). We did not find any effect of Age on the Euclidean distances between dipoles (F(1,96) = 1.53, p = 0.22). The localization discrepancies between Pipeline 2 with the generic head model and Pipeline 4 with the anatomically accurate head model were similar between younger and older adults (12.9 [2.5] mm vs. 13.4 [4.4] mm; t(96) = 2.1, Bonferroni corrected p = 0.12 when the skull conductivity was 0.01S/m; Fig. 7b). Similarly, localization discrepancies between the most simplified forward modeling Pipeline 1 with generic head model and template electrode locations and Pipeline 4 were similar between younger and older adults (21.6 [7.1] mm vs. 18.1[8.0]mm; t(96) = −2.2, Bonferroni corrected p = 0.081; Fig. 7a).

### C. Sensitivity of Conductivity Values

**Fig. 8.**
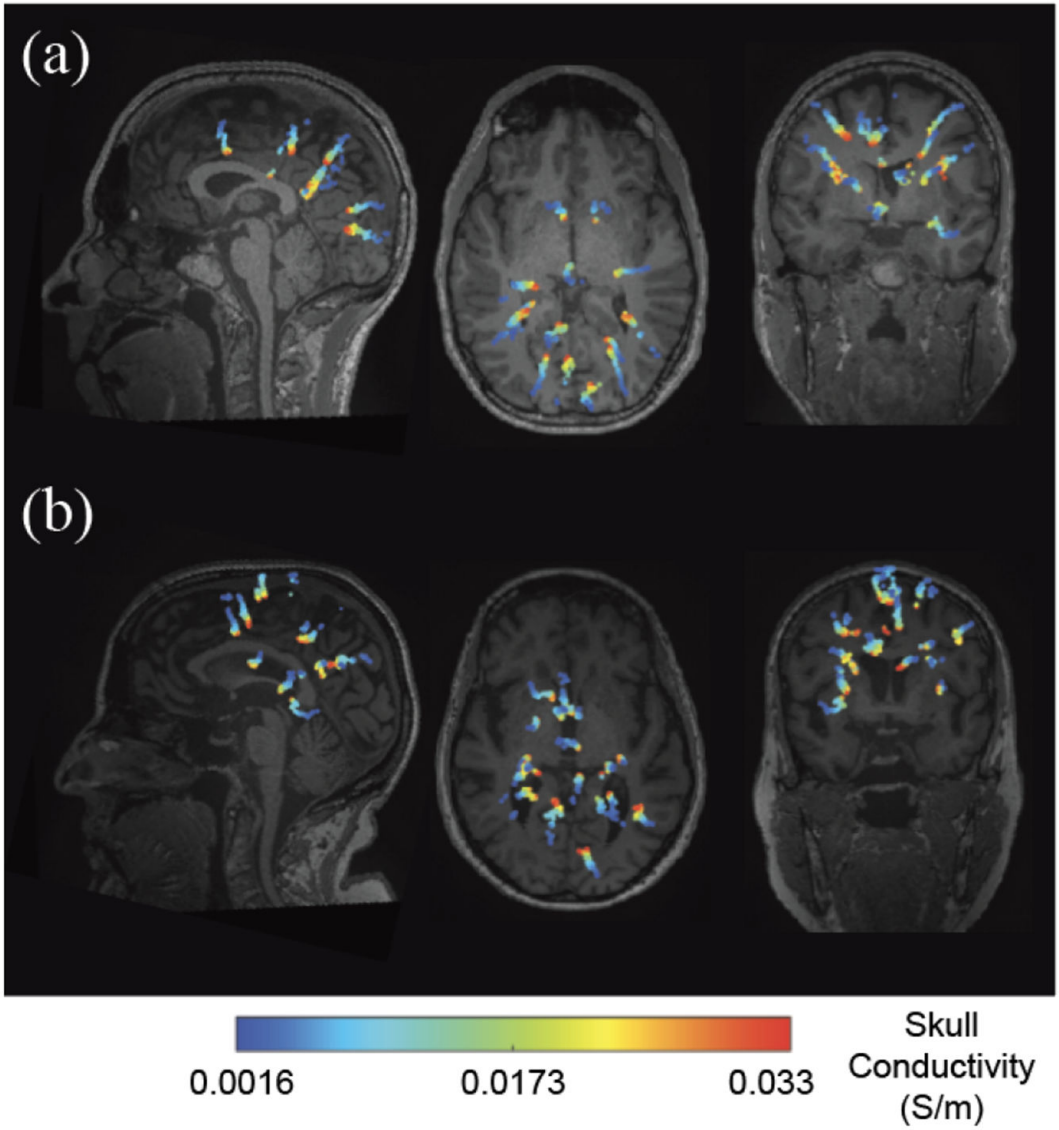
Visualization of brain source locations changing with skull conductivity for one representative younger (a) and older (b) participant on the T_1_-weighted MRIs using Pipeline 4. Color bar ranges from low skull conductivity (blue) to high conductivity (red). Descriptively, source depth increased with skull conductivity value.

**Fig. 9.**
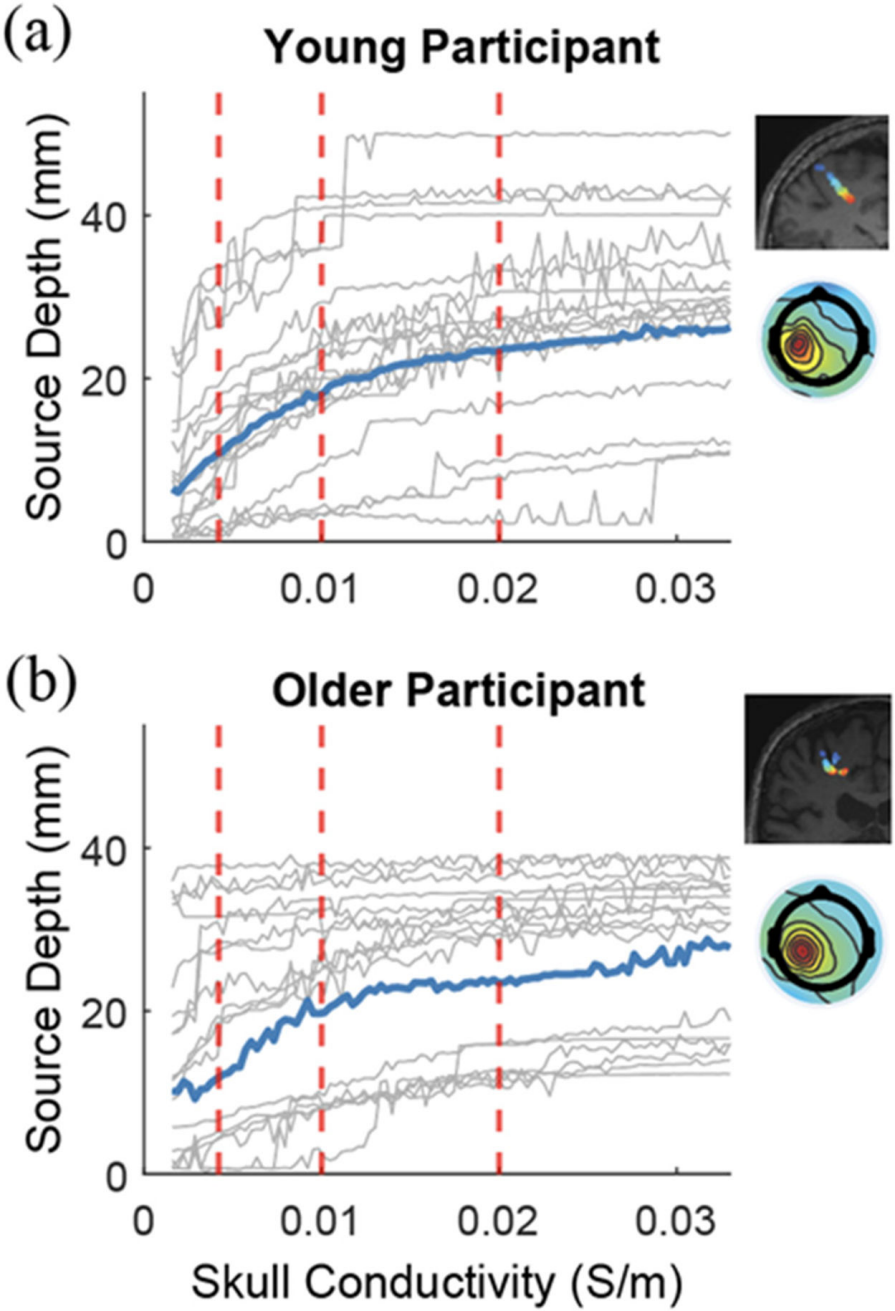
Source depth changed with skull conductivity in one representative younger (a) and older (b) participant. Each gray line indicated one brain component. Blue lines indicate one example brain component located near sensorimotor cortex for both the younger and older adult. Scalp topography and visualization of brain source locations changing with skull conductivity for the highlighted sensorimotor component were presented on the right side. Red vertical dashed lines indicate the skull conductivity values (0.0042S/m, 0.01S/m, 0.02S/m) chosen in this paper.

Dipole locations showed systematic differences with variation in the skull conductivity (in the range of 0.0016 – 0.033 S/m) for the randomly selected three younger adults and three older adults using Pipeline 4 (Fig. 8-9, Supplementary Fig. 3-4 for all six participants). In general, source depth increased with skull conductivity values (Fig.9, Supplementary Fig. 4). Since the most prominent age differences in gray matter volume and thickness occur in sensorimotor cortices [20], [58], we highlighted the brain sources near the sensorimotor cortex (Fig.9 Blue trace). The depth of the brain source at the sensorimotor area increased sharply until ~0.01 S/m for all participants. Source depth increased slowly when skull conductivity increased from 0.01 S/m to 0.033 S/m.

### D. Comparison of Computation Time

Reasonable computation time could be achieved with parallel processing (Table III). For Pipeline 3 and Pipeline 4, the most time-consuming steps were computing the transfer matrix (*ft_prepare_vol_sens*) and dipole fitting (*ft_dipolefitting*) as previously reported in Vorwerk et al. [53]. Computation time for both steps could be greatly improved if implementing parallel processing in MATLAB with *parfor*. Using 20 CPUs with 8 GB/CPU takes less than an hour to create the forward head model.

## IV. Discussion

The primary objective of this study was to determine the errors in source localization caused by using simplified generic head models versus anatomically accurate individual-specific head models based on MRIs in both younger and older adults. To perform a full analysis of forward modeling errors on source estimations, we also quantified source localization discrepancies introduced by inexact electrode locations, inaccurate tissue segmentation, and skull conductivity values. We extracted independent components from a dataset using high-density EEG for both younger and older adults performing walking tasks and motor imagery. We performed dipole fitting to obtain source locations following forward modeling pipelines with increasing complexity. Consistent with our hypothesis, simplified generic head models led to large source localization discrepancies (up to 2cm) compared to the most anatomically accurate model for both younger and older adults. Inexact electrode locations led to an increase in localization discrepancies by an average of 6 mm compared to using digitized electrode locations. Performing a simplified tissue segmentation that assumed constant skull thickness also led to ~6 mm of source localization discrepancies compared to implementing an anatomically accurate segmentation. Contrary to our hypothesis, source localization discrepancies between the generic head models and the most anatomically accurate individual-specific head models were similar between younger and older adults. In addition, we performed a sensitivity analysis to assess how skull conductivity affected brain source location. We found that the depth of estimated sources generally increased with skull conductivity for both younger and older adults, especially when skull conductivity was <0.01 S/m. These results demonstrated how the use of generic head models, inexact electrode locations, simplified tissue segmentation, and skull conductivity affected EEG source localization. Overall, a cautious interpretation of EEG source location is needed when using a simplified generic head model to perform dipole source localization.

We found large differences in the source location (up to 2 cm) between the simplified generic head model and the anatomically accurate head model, which were consistent with the previous literature [4]. Several factors could contribute to the source localization differences between using a generic head model and an individual-specific head model. For example, the three-layer (scalp, skull, brain) generic head model used in the present study did not include a cerebrospinal fluid layer. Thus, the generic three-layer head model can distort EEG potentials and dipole source localization as the cerebrospinal fluid has the highest conductivity value among all tissues [26]. An average of 4 mm differences in source localization were reported with and without accounting for cerebrospinal fluid in the head model [59]. Although co-registering digitized electrode locations improved source localization, an average of 13 mm localization discrepancies were still present. One explanation is that digitized electrode locations could not be warped perfectly to the generic head model as co-registering digitized electrodes to the head model only allowed nine degrees of freedom (scaling, translation, rotation) in DIPFIT toolbox but not a more precise non-linear warping method.

### A. Age Effects on Localization Difference

Contrary to our hypothesis, source localization differences between the generic head models and individual-specific head models were similar in younger and older adults, despite larger variations in brain structure compared to the template MRIs in older adults. One potential explanation between our hypothesis and the observed results is that changes in the electrical properties of the head with aging may have small effects on EEG source locations (in millimeters) and may become unlikely to distinguish from the effects of simplified head model (in centimeters). A prior study that performed a forward simulation analysis suggested that a 10% cortical shrinkage only led to EEG power reduction by ~3.7 dB [19]. In comparison, older adults demonstrated up to ~20 dB power reduction relative to younger adults during resting trials [60]. These results suggest that changes in the electrical properties of the head may have small effects on EEG signals. Thus, although cerebrospinal fluid fills up the space where there is cortex atrophy in older adults, such changes in the electrical properties of the head may also have a small effect on source locations (on the scale of millimeters) and become unlikely to detect. Our analysis suggests that although individual-specific head models may improve the source localization compared to the simplified generic head model, there were no additional benefits of using individual-specific head models for older adults versus younger adults.

### B. Simplified Tissue Segmentation

Simplified tissue segmentation in individual-specific head models affected source localization. Pipeline 3 used a simplified tissue segmentation method that assumed constant skull thickness and neglected details below the skull. We found an average of 6 mm source localization differences comparing pipelines using simplified tissue segmentation and anatomically accurate segmentation. The simplified skull segmentation was achieved by a dilation of the inner skull and outer brain surface following the FieldTrip-SIMBIO pipeline [53]. Our results were in line with prior work demonstrating that head models with constant skull thickness of 6 mm could lead to 5-10 mm of localization error near the base of the brain when compared to the anatomically accurate reference model [61]. One minor benefit of using simplified tissue segmentation is to save computation time (~2-3 hr/scan) needed to perform the anatomically accurate segmentation. However, with the advances in parallel processing, performing anatomically accurate segmentation will be faster.

### C. Electrode Locations

Co-registering the digitized electrode locations to the generic head model improved the source localization compared with using template electrode locations. Using an infrared 3D scanner to digitize EEG electrodes has shown to be a reliable and cost-effective approach. The infrared 3D scanner is aligned with the iPad camera to generate high-resolution colored 3D images. With the *get_chanloc* toolbox in EEGLAB, digitizing electrode locations relative to fiducial locations can be user-friendly and time efficient, usually taking less than 15 minutes to complete the digitization process. Our results showed that warping accurate electrode locations to the generic head model can improve source localization by 6 mm when individual MRIs are unavailable. Therefore, such an approach should be considered for future EEG studies to improve the accuracy of source localization.

### D. Skull Conductivity

Source depths increased with skull conductivity in both younger and older adults, which is in line with the previous studies [4], [32]. Although the source locations estimated with a variation of skull conductivity values were approximately near similar locations, rapid changes in source depth occurred when the skull conductivity was below 0.01 S/m. Currently, there is no consensus on skull conductivity despite many attempts to provide estimates of skull conductivity [56]. Literature reported the brain-to-skull conductivity ratio to be between 8 and 80 in adults [33], [34], [35], [36], [37], [38]. The brain-to-skull conductivity ratio is commonly set to 80:1 in open-source head models [37]. The 80:1 ratio is higher than most of the existing literature as many studies report the brain-to-skull conductivity ratio to be 15:1 to 42:1 [33], [34], [35], [36], [38], which is more similar to our choice of 0.01 S/m and 0.02 S/m in this current analysis. In addition, most of the current head models (including in the present study) do not consider the inter-subject variability of skull conductivity values and use the same conductivity across participants and age groups, despite evidence that skull conductivity can decrease with age [39], [40]. Recently, there have been attempts to estimate skull conductivity during source localization to account for the inter-subject variation. For example, Akalin Acar et al. [62], [63] estimated skull conductivity and the location of the EEG sources concurrently using high-density EEG using a novel iterative approach. It remains to be seen whether this novel approach can be adopted to address the age-related changes in skull conductivity.

### E. Limitations

The study had a few limitations. Segmentation was not manually corrected to align appropriately with the exact anatomy of each participant. We chose to follow an automatic tissue segmentation toolbox headreco as it reached high accuracy with reasonable processing time [54]. We did not obtain a T_2_-weighted MRIs, which typically would improve the accuracy of the tissue segmentation, especially for the cerebrospinal fluid. In T_1_-weighted images, cerebrospinal fluid may be mistakenly modeled as bone and lead to an overestimation of skull thickness as the contrast between compact bone and cerebrospinal fluid is not sharp [61]. Future research should examine whether including a T_2_ -weighted scan would change the source localization results. In addition, tissue segmentation was still simplified with the most anatomically accurate pipeline. For example, the geometry of the skull was simplified. Holes that nerves and blood vessels went through were not corrected in skull segmentation. We did not consider other skull perforations, such as parietal foreman which were highly variable across individuals [64]. These holes may result in local errors of about 2 mm around the holes (see simulations in Lanfer et al. [61]). Other simplifications included a generalization of tissue types, such as omitting blood vessels in segmentation. The omission of blood vessels in the forward model could lead to 2 cm source localization error in the deeper brain area, such as the insula or medial temporal lobe [65]. However, detecting blood vessels may require a 7T MR scanner or a black blood sequence to achieve a high signal-to-noise ratio [65], which is currently not feasible for most EEG research. Recently, a new toolbox that uses deep neural networks has shown promising tissue segmentation results with additional tissue classes, such as muscle and blood vessels [66]. Future research may adopt this new toolbox to incorporate more tissue types to improve segmentation accuracy.

Smaller localization differences may be obtained using a template head model with more anatomical details. We chose the three-layer generic head model available in EEGLAB because this head model is widely used in many EEG studies. Template head models with downward extension to include the whole head and neck may help improve source localization at the base of the brain [67], [68]. Future research may evaluate the use of generic head models with more anatomical details and determine the source localization differences compared to the individual-specific head models.

The results of our study are only applicable to source localization method using an equivalent dipole fitting approach. Systematic analysis needs to be performed with various source localization methods (e.g. sLORETA [69], Minimum-Norm Estimation [70]) to help generalize our results to other source localization methods. Another limitation is that there is a lack of ground truth of source locations in our study as we used the most anatomically accurate pipeline as the reference for comparison with other pipelines. Therefore, our findings need to be interpreted with caution and future investigation should incorporate simulated datasets with known ground truth to evaluate the factors contributing to the accuracy of source localization results.

## V. Conclusion

We quantified source localization discrepancies introduced by using generic head models, inexact electrode locations, inaccurate brain region segmentation, and skull conductivity for both younger and older adults. We found that performing dipole fitting with generic head models based on template MRIs led to source localization discrepancies up to 2 cm compared to the anatomically accurate individual-specific head models for both younger and older adults. Overall, these results can provide more insights into the factors that affect EEG source localization and enable a more accurate interpretation of brain areas in EEG studies when individual MRIs are unavailable in both younger and older population.

## Supplementary Material

supp1-3281356

## Figures and Tables

**TABLE I:** Participant Characteristics

Variables	Younger adult median [IQR]^b^	Older adult median [IQR]^b^	P value
Sex	8F, 7M	14 F, 7 M	p = 0.42
Age (years)	22 [4]	74.5 [7.8]	n.a
MoCA	29 [2.5]	28 [2]	p = 0.21
Uneven treadmill walking speed (m/s)	0.76 [0.2]	0.43 [0.4]	p < 0.001
Ventricular Volume (%)^a^	0.60 [0.4]	2.6 [2.2]	p < 0.001
Gray Matter Volume (%)^a^	43.7 [2.4]	36.0 [3.9]	p < 0.001

a Both ventricular volume and gray matter volume are normalized by the total intracranial volume.

b IQR: interquartile range

n.a: not applicable

**TABLE II:** Tissue Conductivity for Each Pipeline

	Pipeline 1	Pipeline 2	Pipeline 3	Pipeline 4
White matter (S/m)	n.a	n.a	0.126	0.126
Gray matter (S/m)	0.33	0.33	0.33	0.33
Cerebrospinal fluid (S/m)	n.a	n.a	1.65	1.65
Skull (S/m)	0.0042	0.0042	0.0042	0.0042
			0.01	0.01
			0.02	0.02
Scalp (S/m)	0.33	0.33	0.33	0.33
Air (S/m)	n.a	n.a	n.a	2.5 × 10^−14^

n.a: not applicable

**TABLE III:** Computation Time for Each Pipeline

	Tissue Segmentation	Forward model	Dipole fitting
Pipeline 1 and 2	n.a	n.a	1 CPU, 15 min
Pipeline 3	1 CPU	20 CPU, 8 GB/CPU memory	16 CPU, 16 GB/CPU
	15 min	~30 min	~45min
Pipeline 4	1 CPU	20 CPU, 8GB memory	16 CPU, 16 GB/CPU
	~2hr	~lhr	~lhr

n.a: not applicable
