# Supplementary material for: Comparison of EEG Source Localization Using Simplified and Anatomically Accurate Head Models in Younger and Older Adults
Source: IEEE Trans Neural Syst Rehabil Eng. Author manuscript; Available in PMC 2023 Jul 12. (PMC10336858; doi:10.1109/TNSRE.2023.3281356)

**Supplementary Table 1:** Statistical results examining the effects of Pipeline and Age on Euclidean distance of dipole locations for each skull conductivity.

| Skull conductivity | Factor | DF1 | DF2 | F value | p value |
| --- | --- | --- | --- | --- | --- |
| 0.0042 S/m | Pipeline | 1 | 96 | 46.9 | <0.0001 |
|  | Age | 1 | 96 | 2.2 | 0.14 |
|  | Pipeline:Age | 1 | 96 | 3.1 | 0.05 |
| 0.01 S/m | Pipeline | 1 | 96 | 67 | <0.0001 |
|  | Age | 1 | 96 | 1.5 | 0.22 |
|  | Pipeline:Age | 1 | 96 | 5.3 | 0.007 |
| 0.02 S/m | Pipeline | 1 | 96 | 122 | <0.0001 |
|  | Age | 1 | 96 | 0.6 | 0.44 |
|  | Pipeline:Age | 1 | 96 | 6.3 | 0.003 |

**Supplementary Fig. 1:** Number of independent components labeled as brain with different preprocessing parameters for a subset of young (n=15) and older adults (n=15) for Mind in Motion study. Each dot represents one participant. Black squares indicate the average number of brain components by ICLabel for each preprocessing parameter selection. Chan: Chan_crit1, Win: Win_crit1.

**
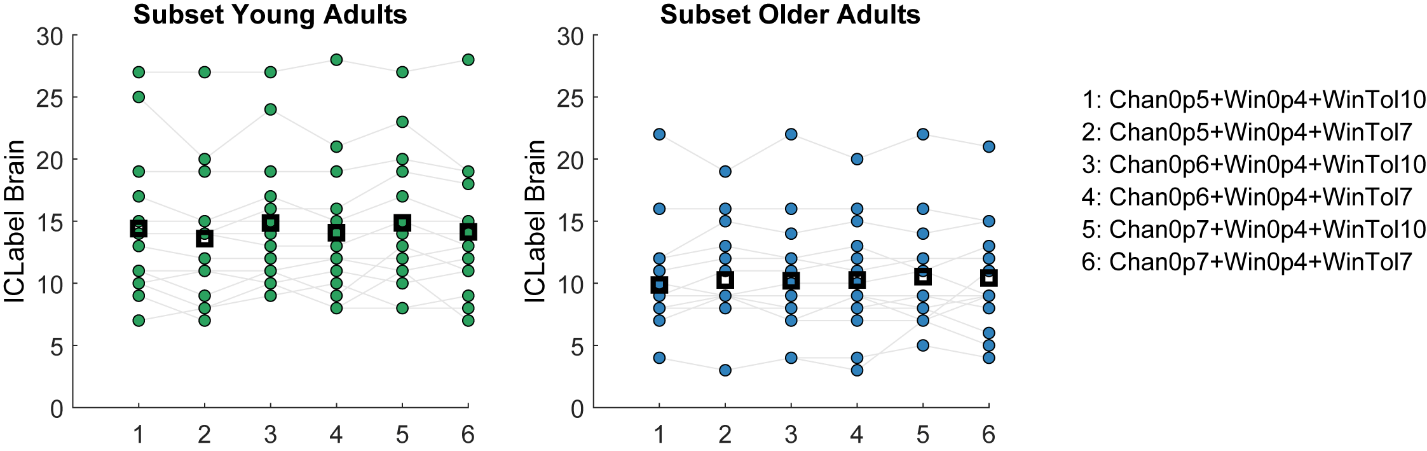
**

We chose the preprocessing parameters based on the preprocessing outcome from a subset of data from the large Mind in Motion study. The subset of data included 15 young participants and 15 older adults. We carefully selected the following parameters for pre-processing 1) rho^2^ for iCanClean algorithm that removed mutually correlated components between EEG data and noise data, 2) chan_crit1, 3) win_crit1), and 4) winTol in *clean_artifacts*.

For the rho^2^ in iCanClean algorithm, we have recently published a paper on parameter selection and one set of optimal parameter is rho^2^  = 0.9 combined with a two-second cleaning window to obtain the maximum number of brain components labeled by ICLabel (Gonsisko et al., 2023).

In addition to iCanClean, we used *clean_artifact* to reject bad EEG channels and time frames. We first counted the number of channels rejected by performing a parameter sweeping with [0.5, 0.6, 0.7, 0.75, 0.8, 0.85] for chan_crit1 on the subset of the data. Since we wanted to retain >90% of EEG channels for most of our participants, we found that chan_crit1 would need to be ≤ 0.7. We then performed a parameter sweep with [0.25 0.3 0.35 0.4] for win_crit1. Similarly, we found that using win_crit1 of 0.4 would make sure that the percentage of time frame rejected would be below 10% for most of our participants in the subset.s

Lastly, we tested how combinations of these clean_artifacts parameters (chan_crit1, win_crit1, and winTol) would change the number of brain components. We tested six combinations (chan_crit1 = [0.5, 0.6, 0.7], win_crit1 = 0.4, winTol = [7, 10]) (Figure 1). We found that using stricter criteria does not significantly increase the number of brain components labeled by ICLabel. Therefore, we chose 0.5 for chan_crit1, 0.4 for win_crit1, and 10 for win_Tol for this current analysis.

**Supplementary Fig. 2:** Dipole locations estimated by four forward modeling pipelines with increasing complexity warped to a template MRI in MNI space and representative brain components for all participants (n = 15 younger adults, n = 19 older adults). Yellow dots: dipoles estimated using Pipeline 1; Green: Pipeline 2; Red: Pipeline 3; and Blue: Pipeline 4. Dipoles that were grouped together were estimated using the same brain component. Note that two dipole locations estimated using Pipeline 2 were outside of head. We still kept those in our analysis as the same dipoles estimated using other pipelines were still in the head.


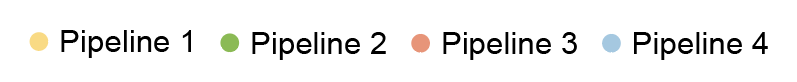


**Younger adults**


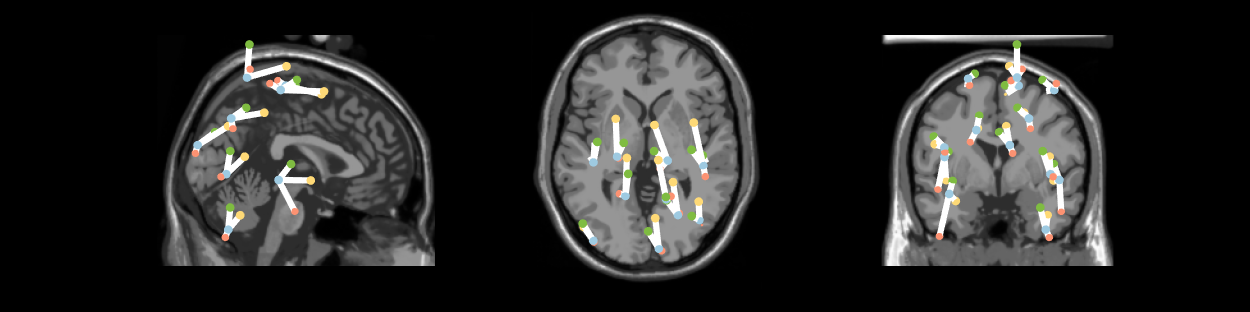

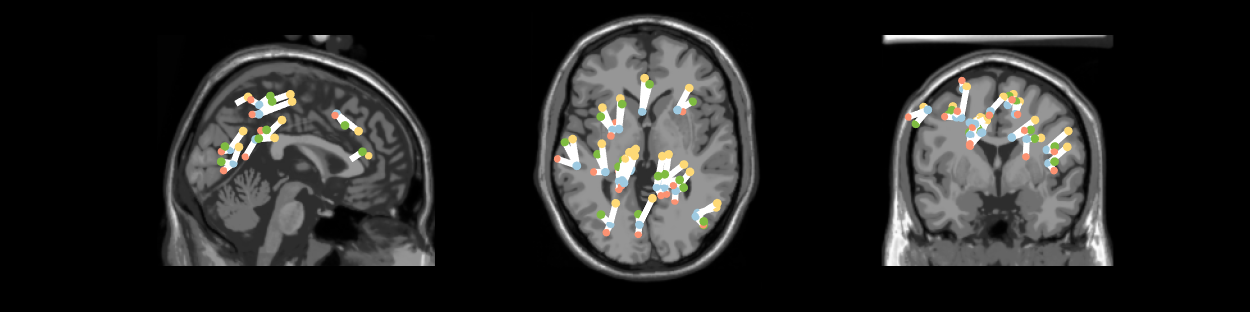

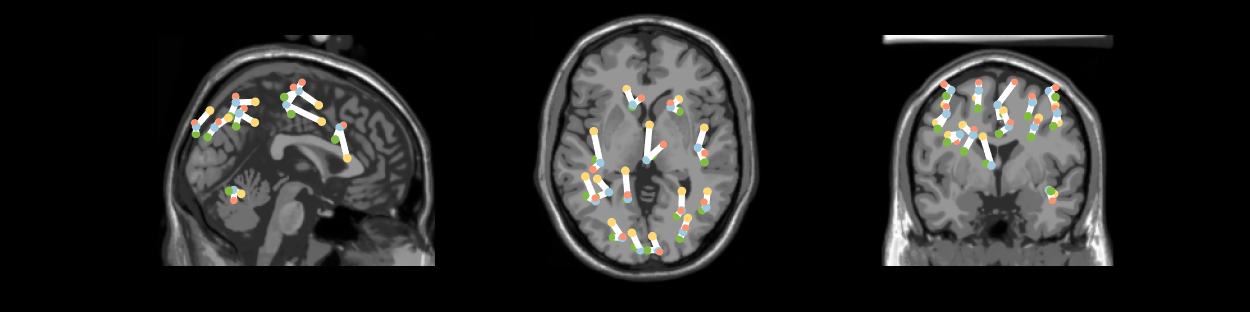

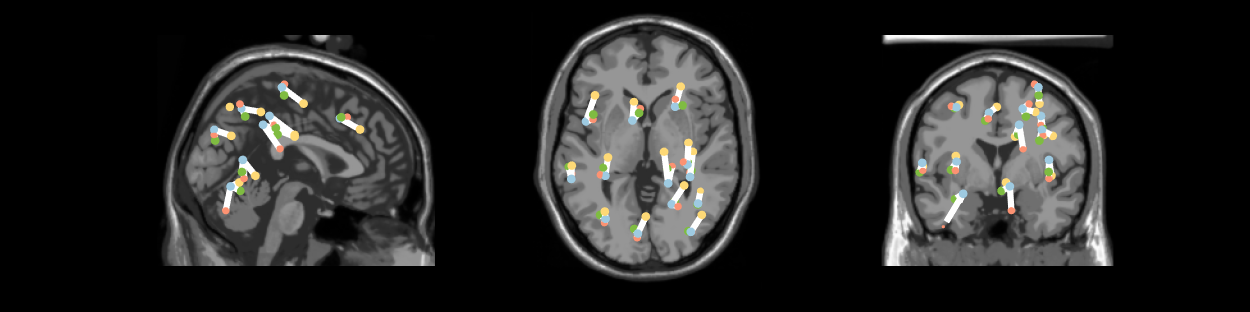

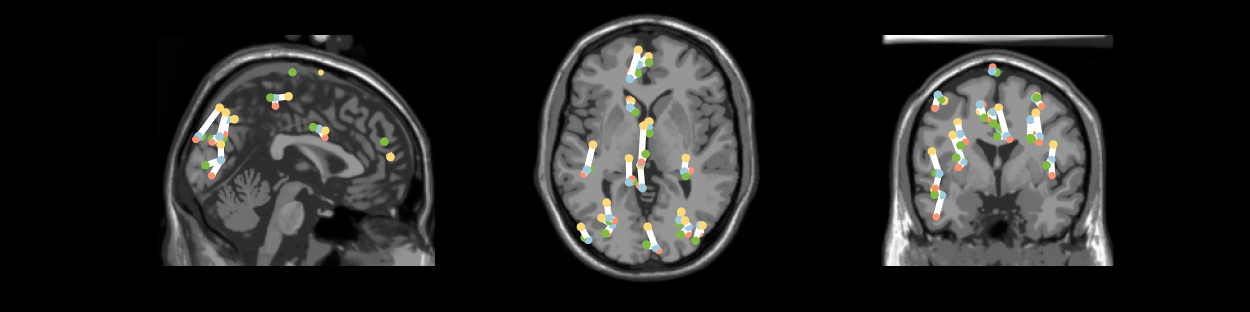

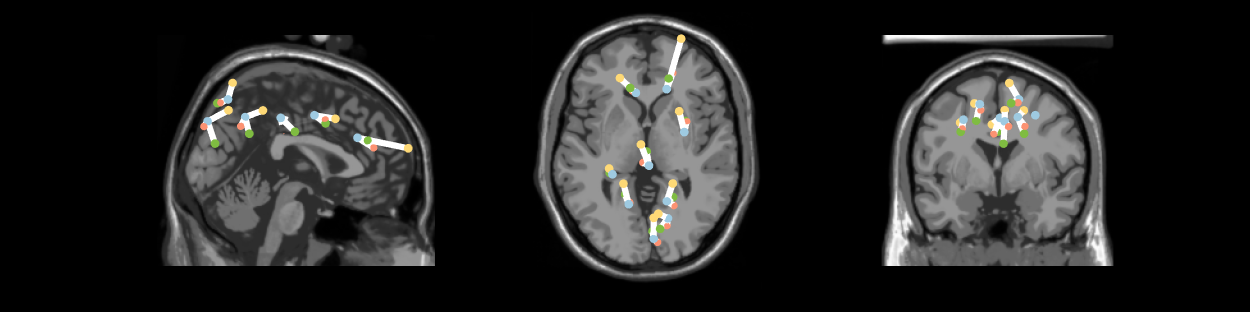

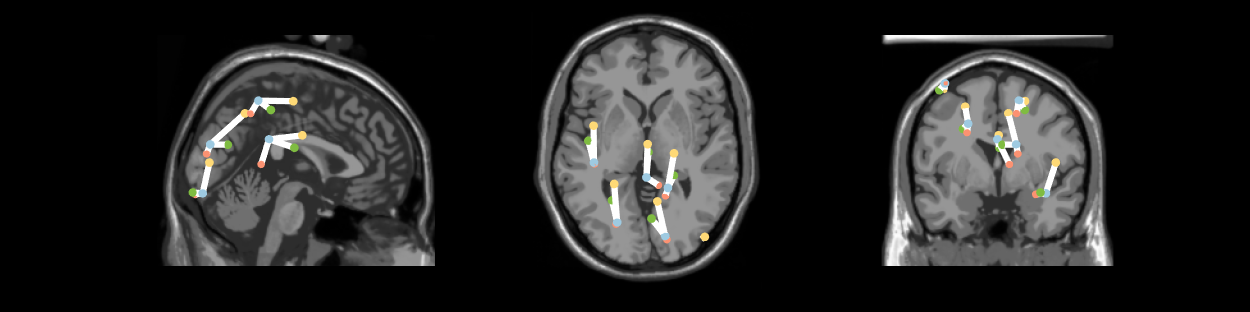

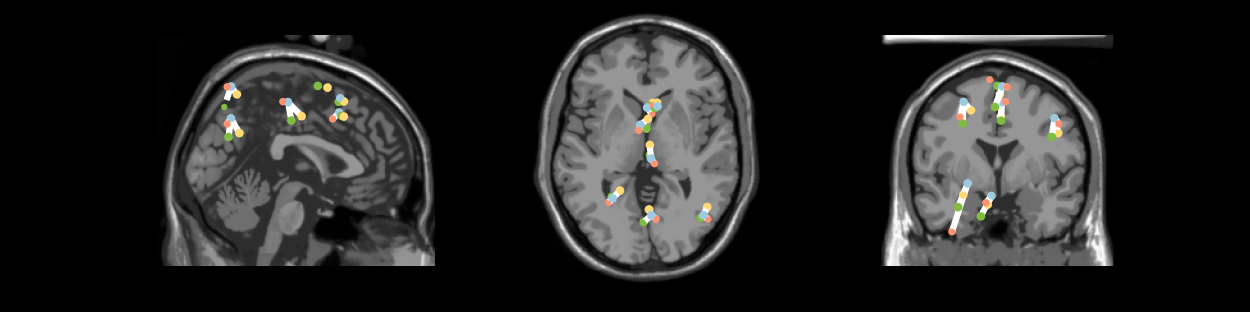

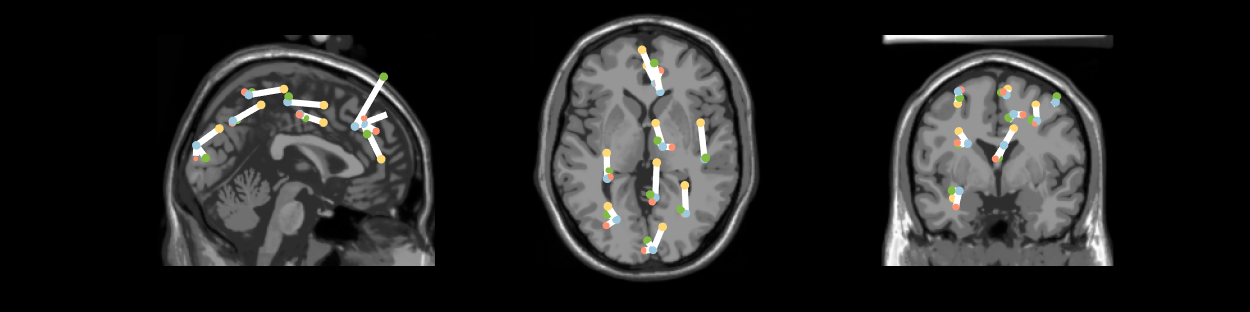

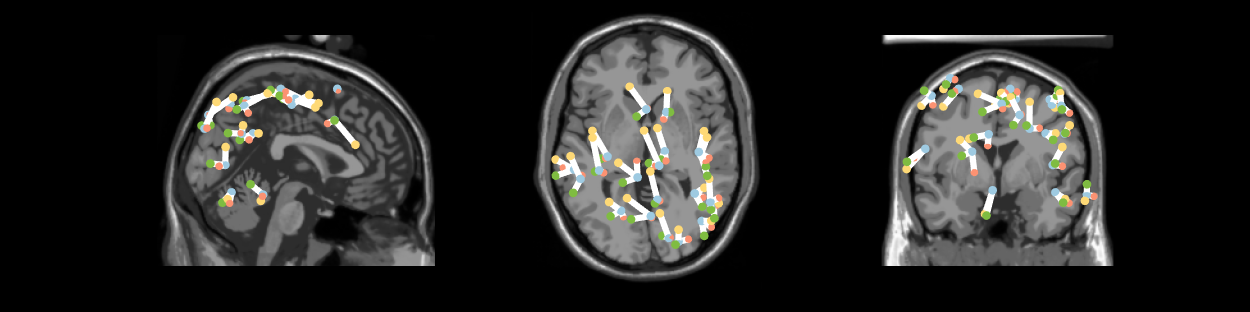

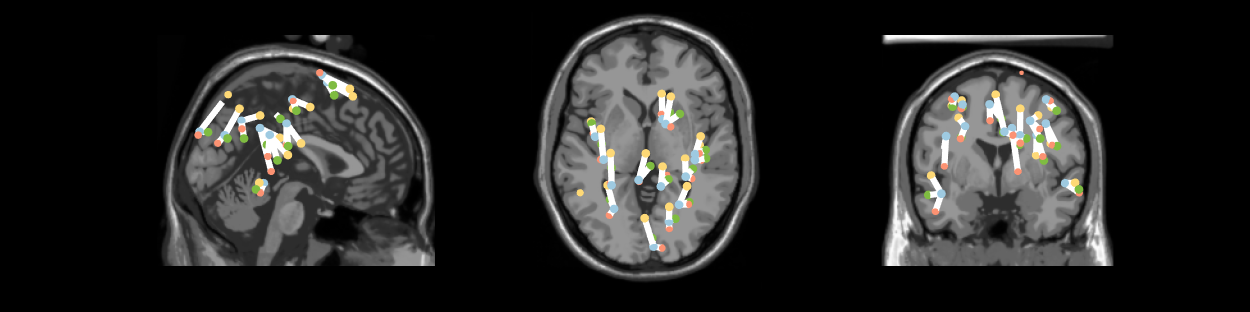

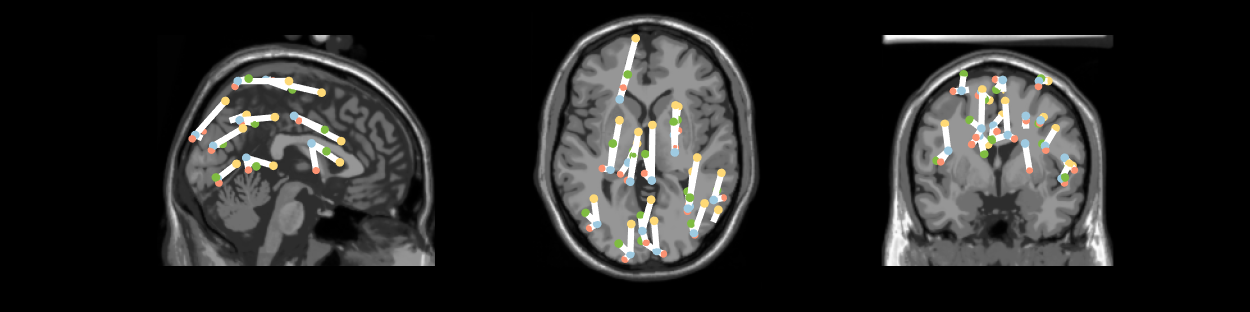

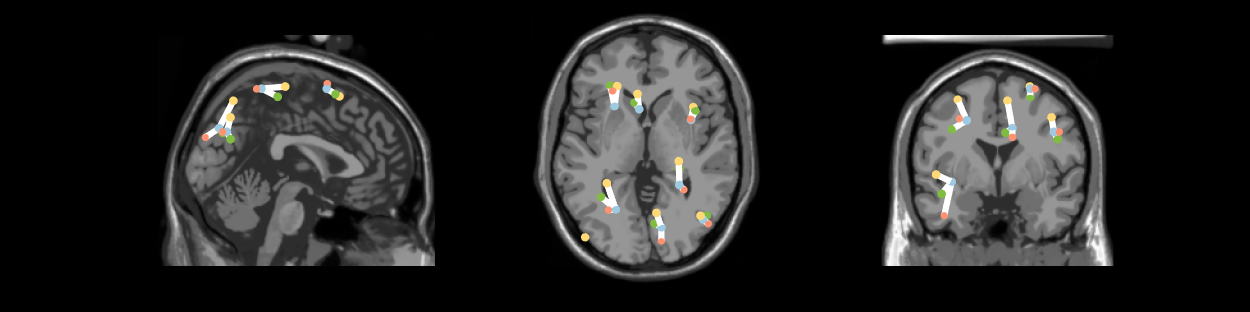

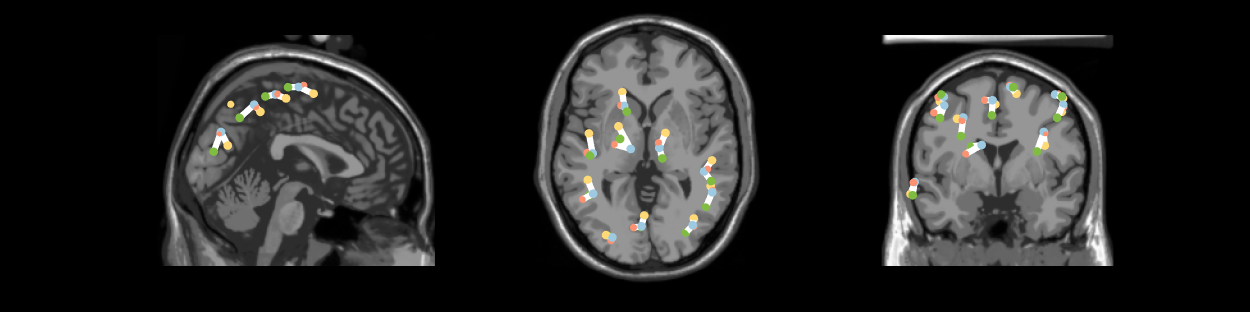

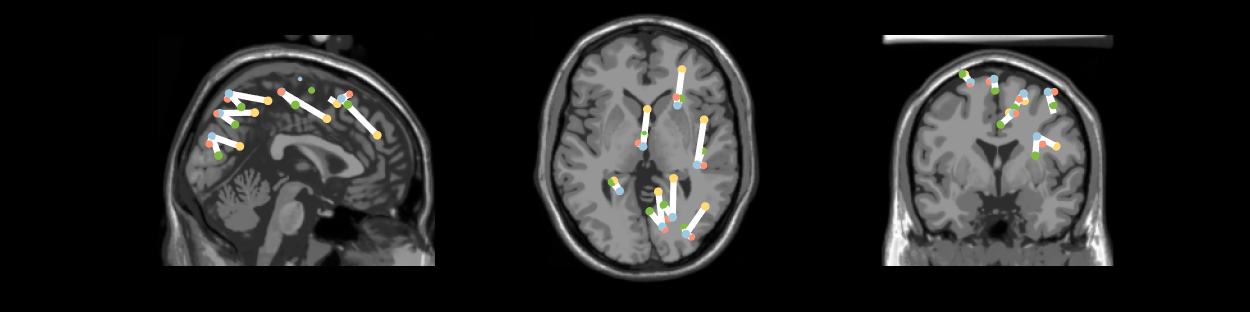


Older adults


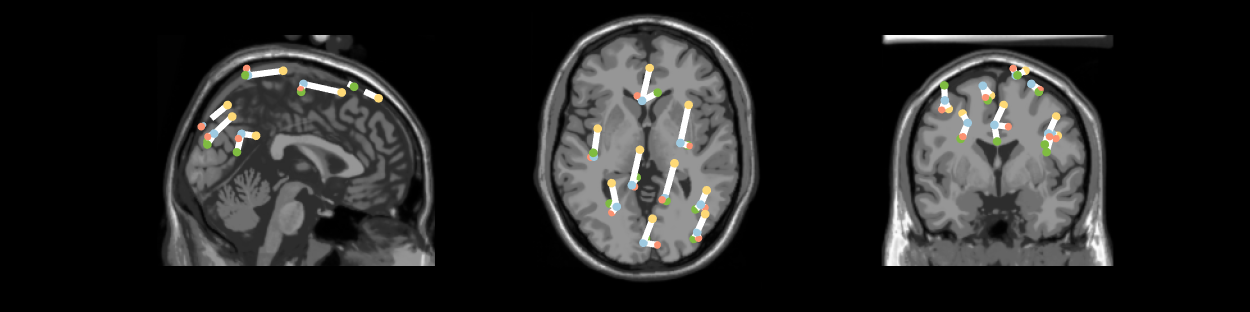

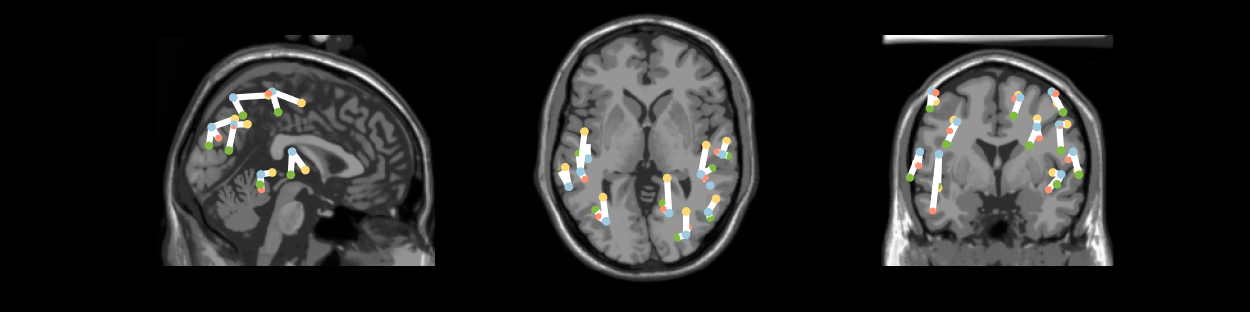

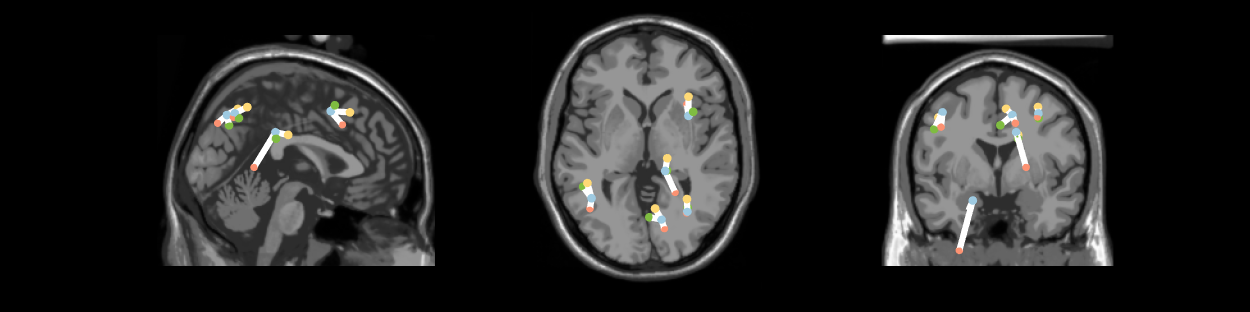

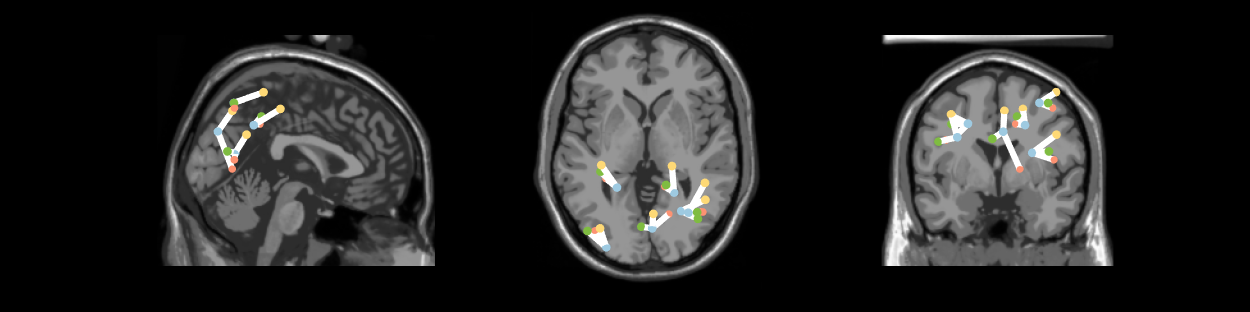

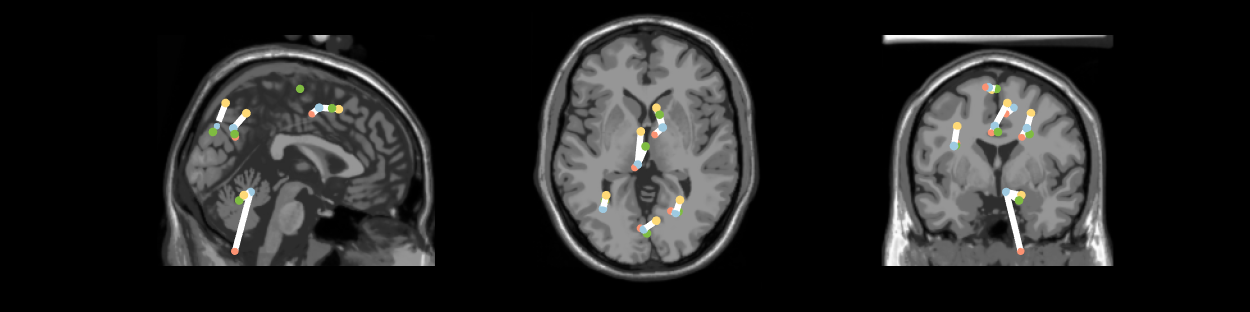

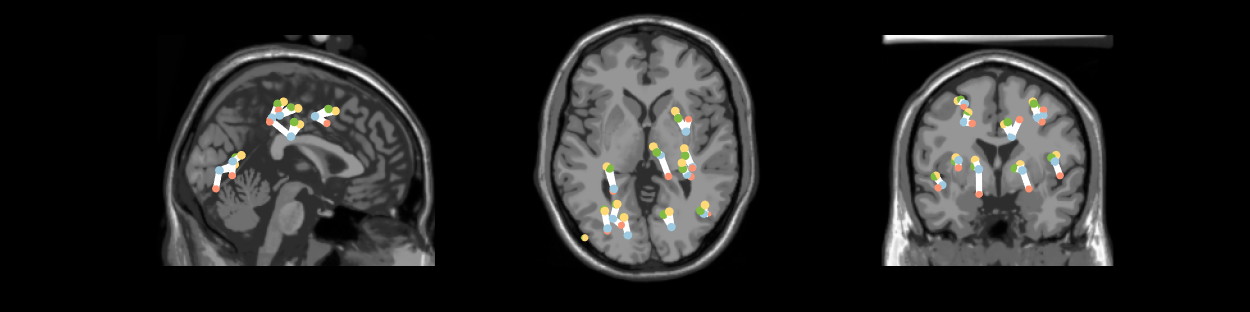

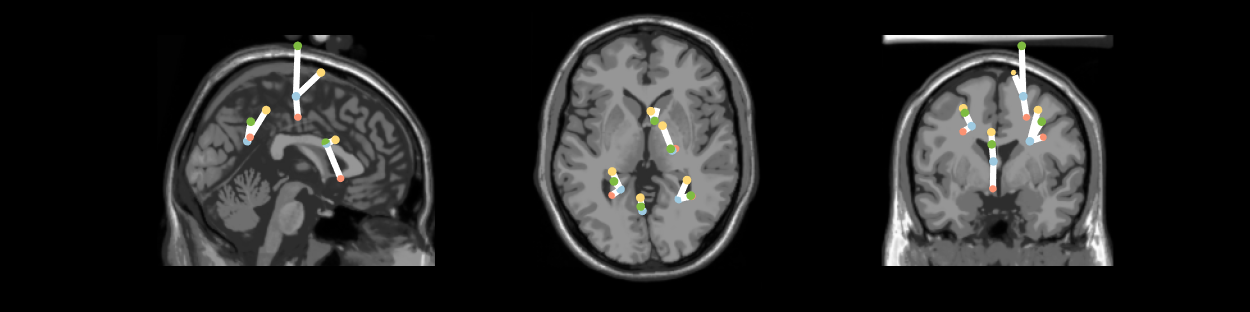

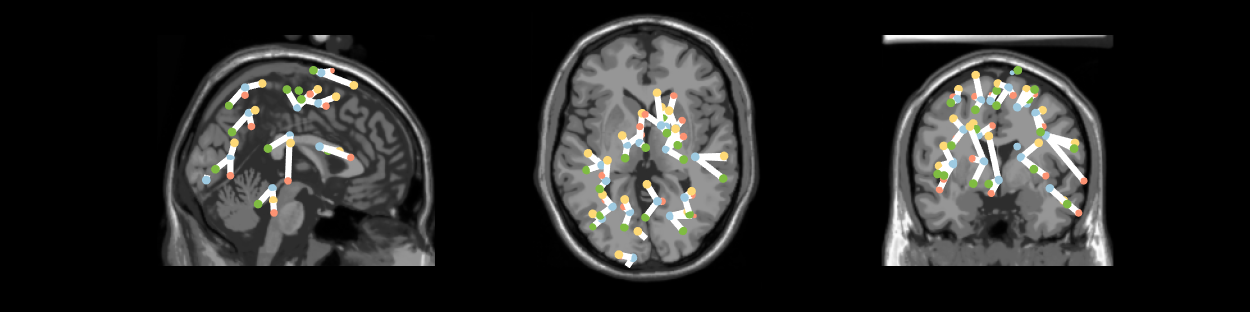

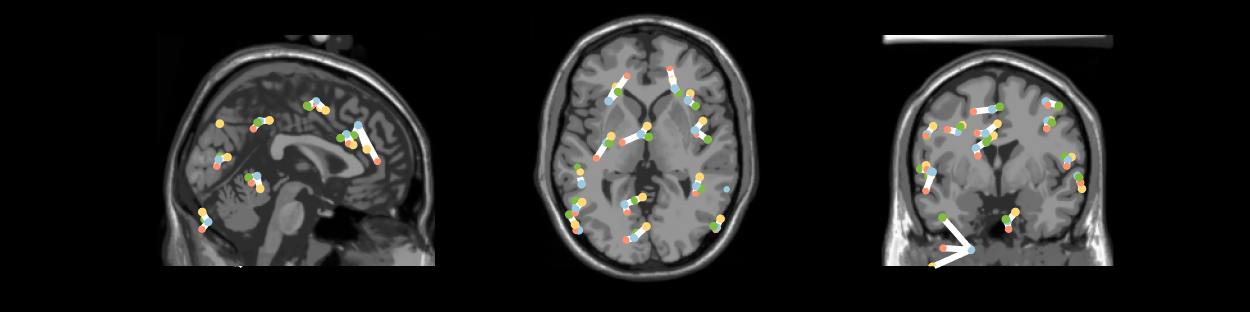

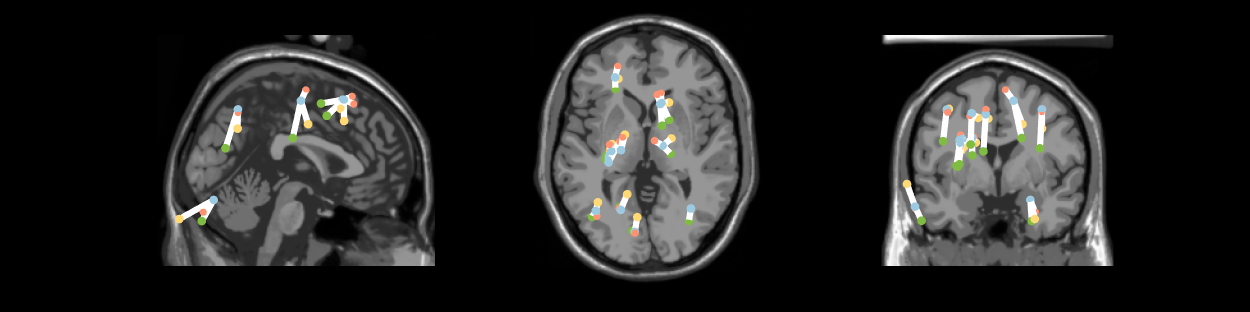

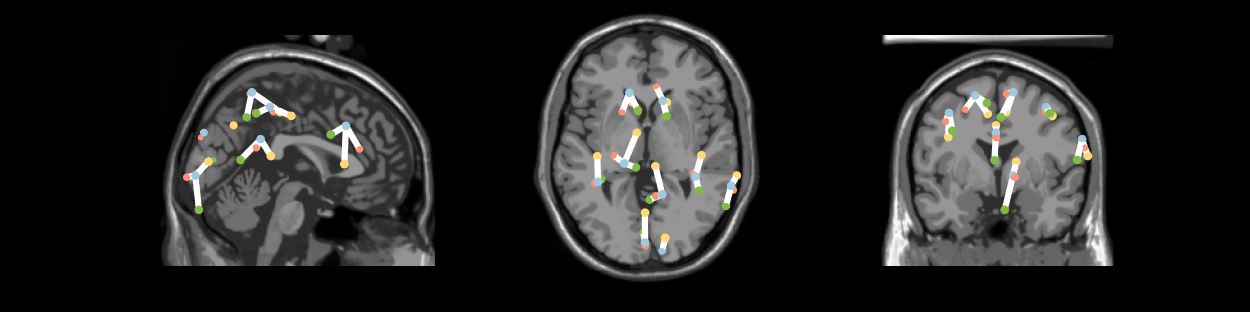

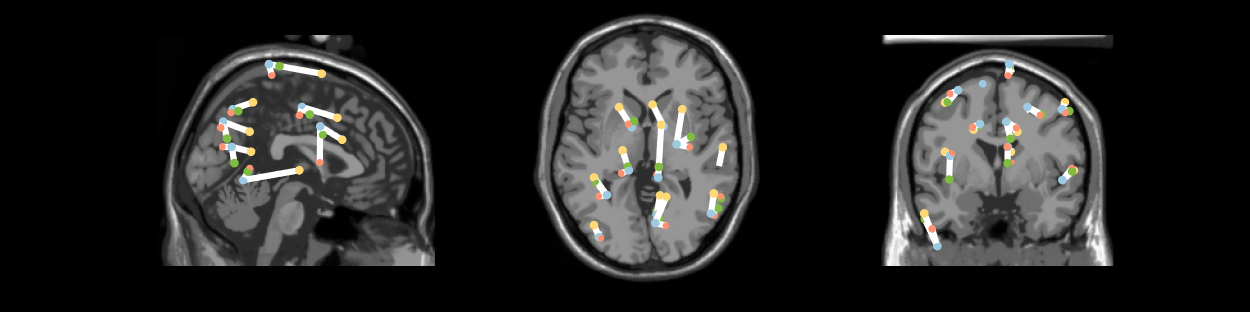

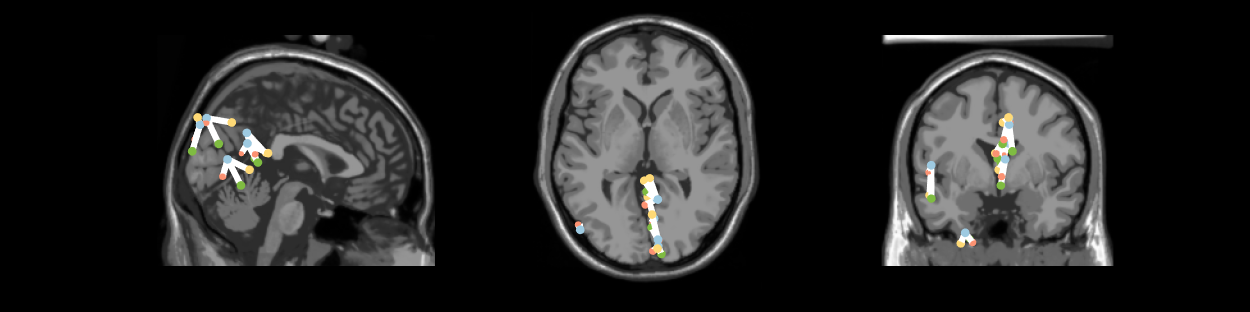

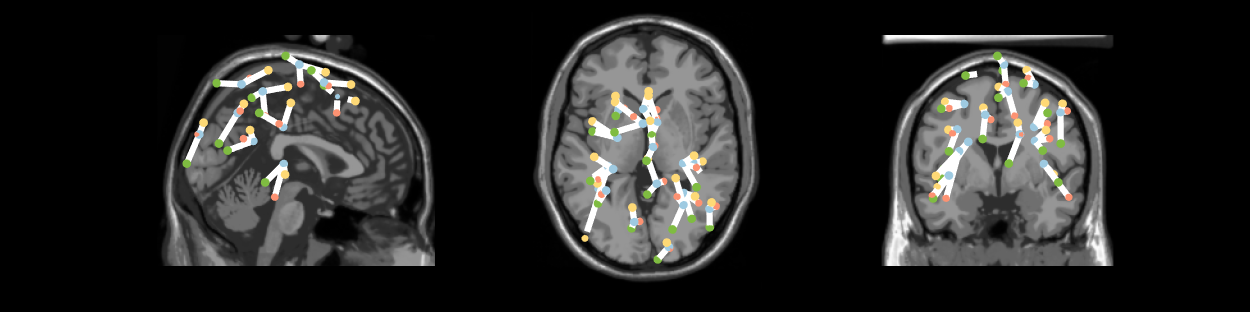

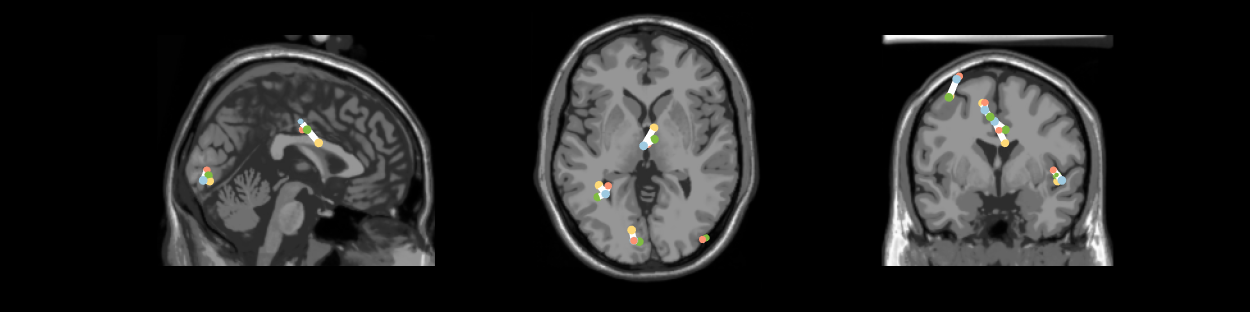

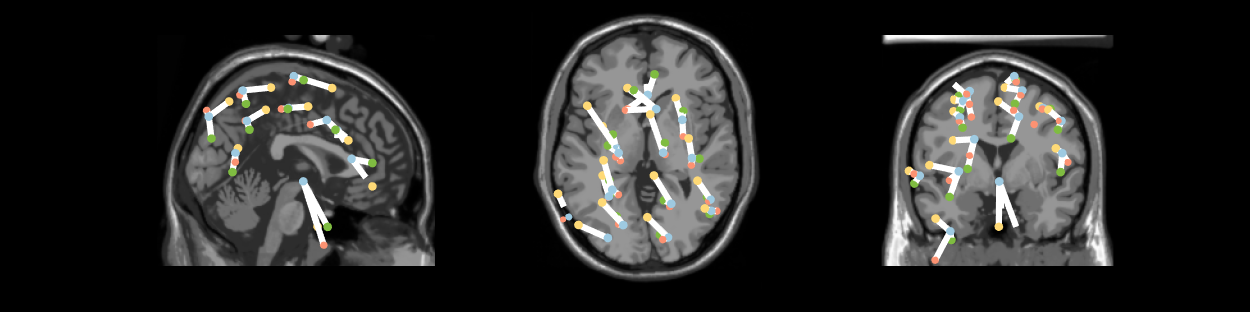

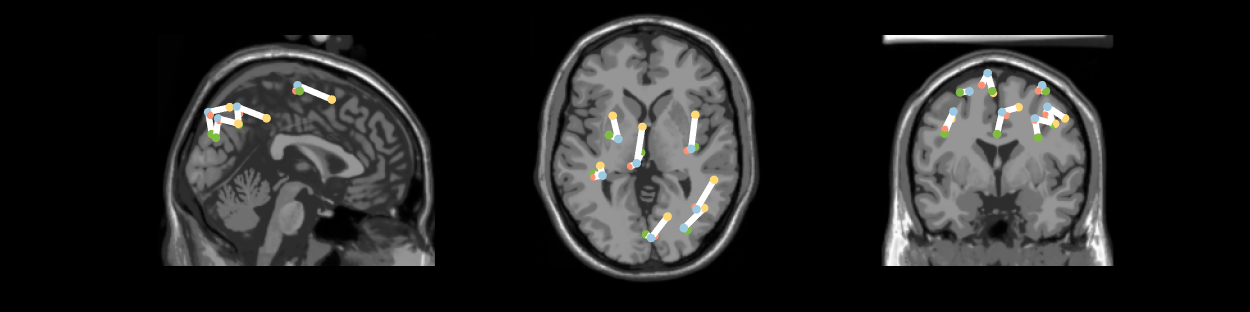

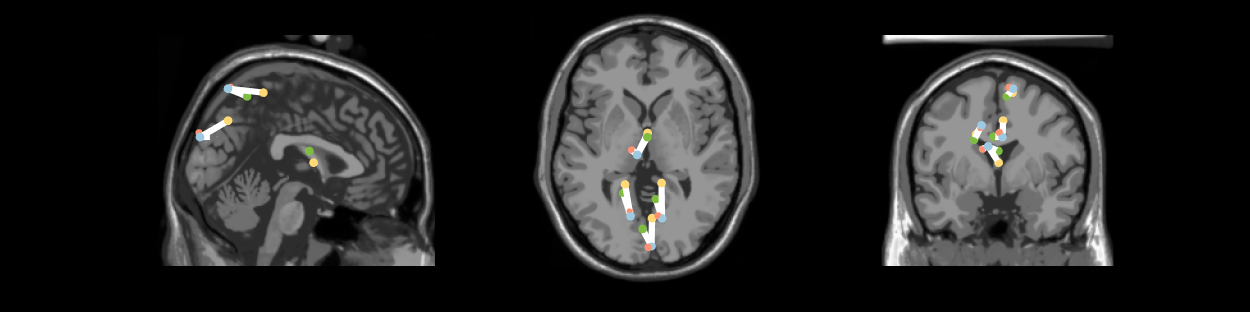

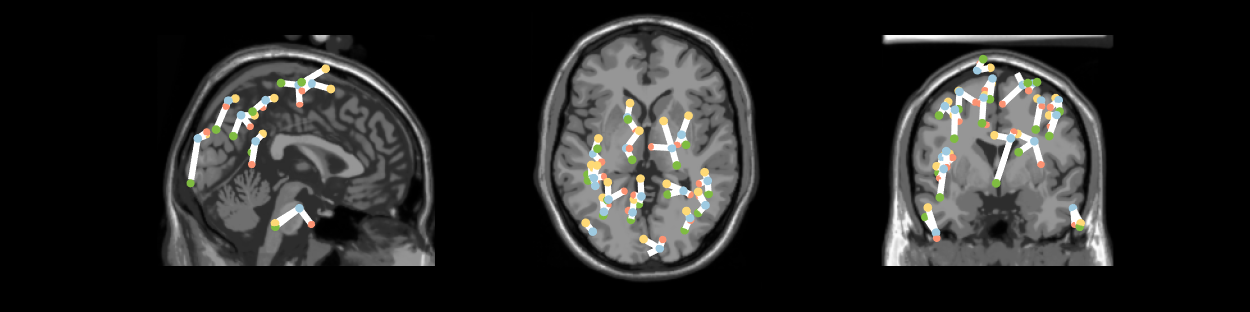


**Supplementary Fig. 3:** Visualization of brain source locations changing with skull conductivity for three young (a-c) and older (d-f) adults on the T1-weighted MRIs using Pipeline 4. Color bar ranges from low skull conductivity (blue) to high conductivity (red). Descriptively, source depth increased with skull conductivity value.


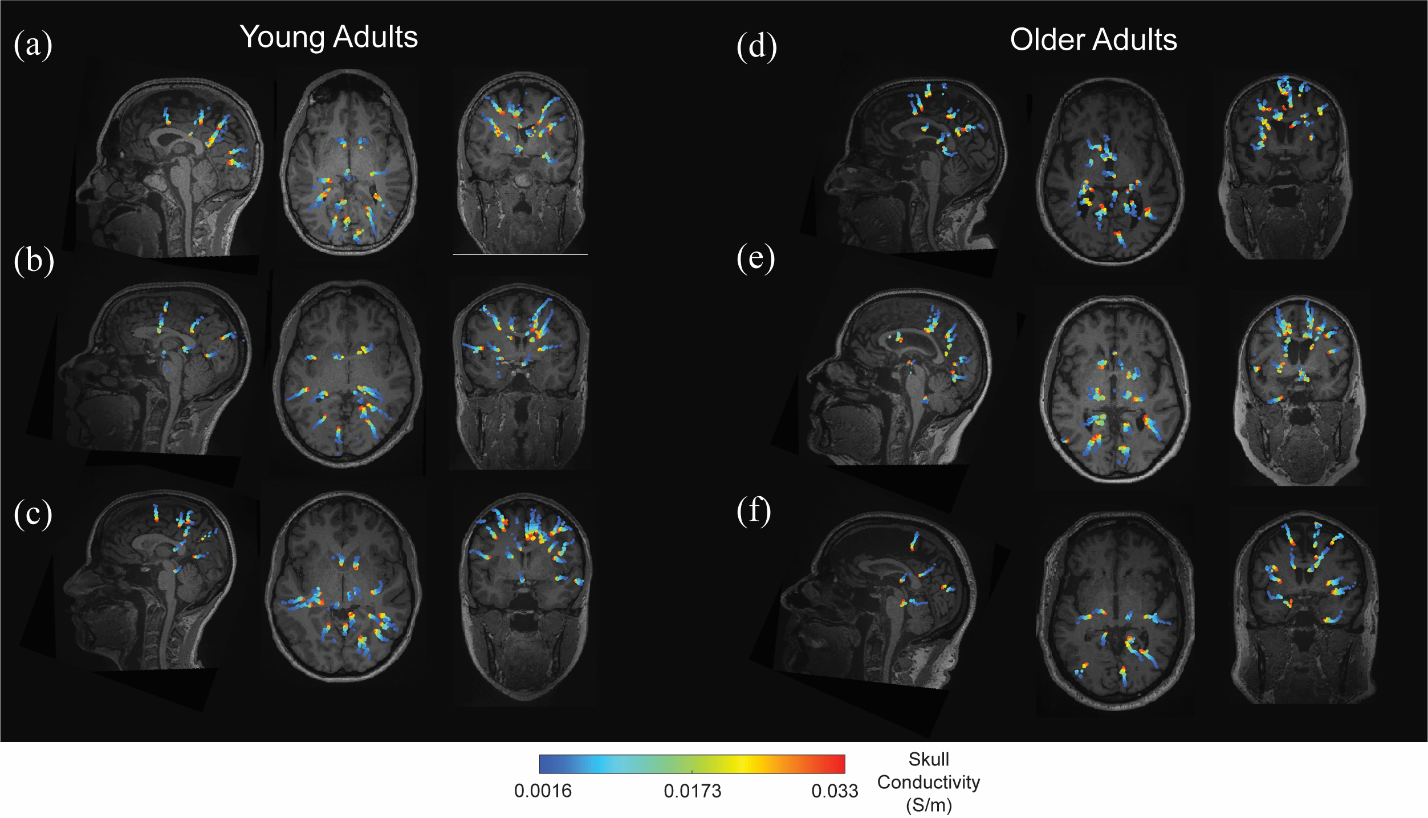


**Supplementary Fig. 4:** Source depth changed with skull conductivity in three young (a-c) and two older (d-f) participants. Each panel represents one participant. Each gray line indicated one brain component. Blue lines indicate one example brain component located near sensorimotor cortex. Scalp topography and visualization of brain source locations changing with skull conductivity for the highlighted sensorimotor component were presented on the right side. Red vertical dashed lines indicate the skull conductivity values (0.0042S/m, 0.01S/m, 0.02S/m) chosen in this paper.


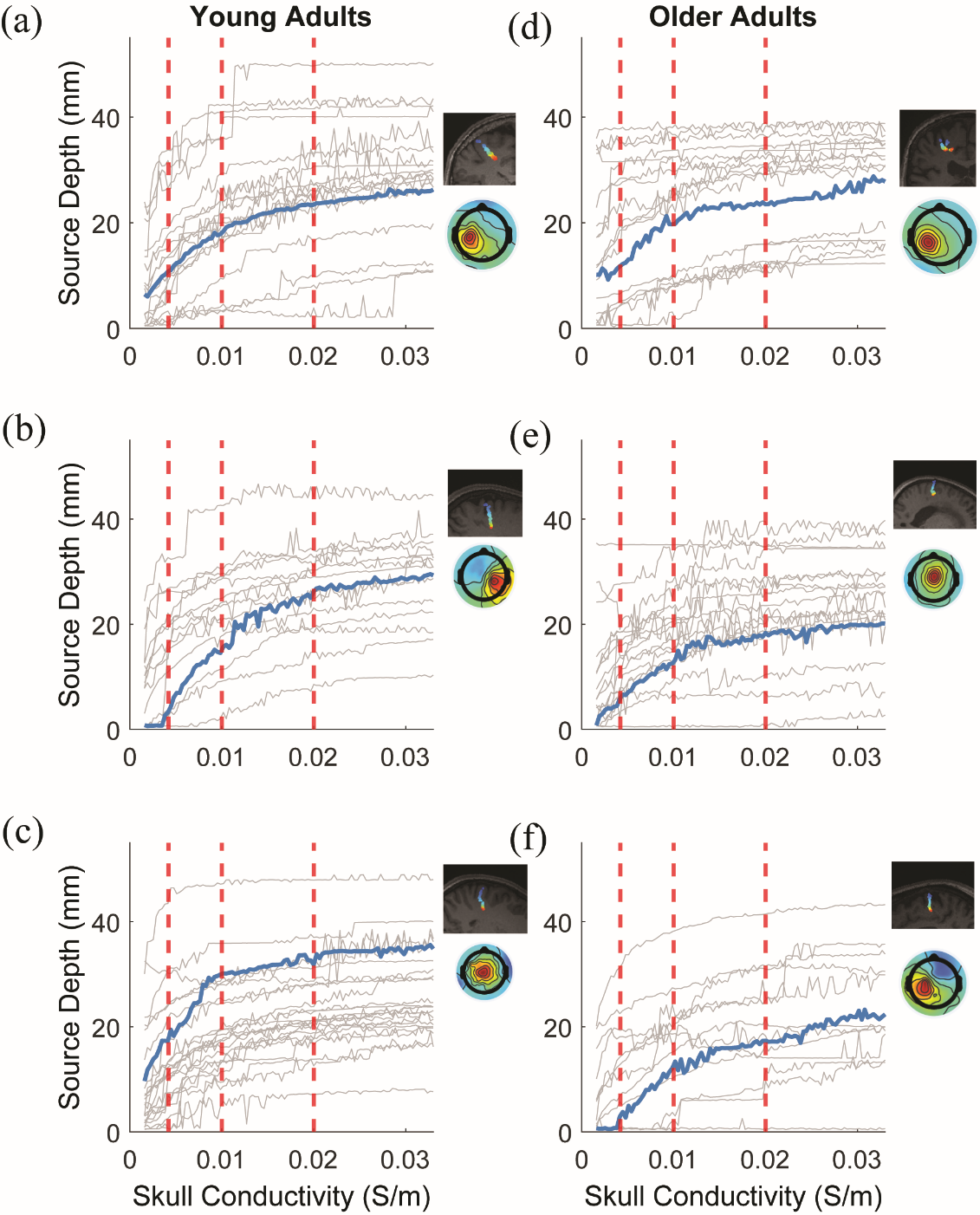

Supplement: supp1-3281356 [file NIHMS1908522-supplement-supp1-3281356.docx]
